# Recent Progress and Future Prospects of Anions O‐site Doped Perovskite Oxides in Electrocatalysis for Various Electrochemical Systems

**DOI:** 10.1002/advs.202304224

**Published:** 2023-10-31

**Authors:** Caichen Yang, Yunfeng Tian, Chenghao Yang, Guntae Kim, Jian Pu, Bo Chi

**Affiliations:** ^1^ State Key Laboratory of Material Processing and Die & Mould Technology School of Materials Science and Engineering Huazhong University of Science and Technology Wuhan 430074 China; ^2^ Jiangsu Key Laboratory of Coal−based Greenhouse Gas Control and Utilization School of Materials Science and Physics China University of Mining and Technology Xuzhou 221116 China; ^3^ Key Laboratory of Interfacial Physics and Technology Shanghai Institute of Applied Physics Chinese Academy of Sciences Shanghai 201800 China

**Keywords:** anion doping, electrocatalysis, O‐site, perovskite oxides, prospectives

## Abstract

With the rapid development of novel energy conversion and storage technologies, there is a growing demand for enhanced performance in a wide range of electrocatalysts. Perovskite oxides (ABO_3_) have caused widespread concerns due to their excellent electrocatalytic properties, low cost, stable and reliable performance. In recent years, the research on anion O‐site doping of perovskite oxides has been a cynosure, which is considered as a promising route for enhancing performance. However, a systematic review summarizing the research progress of anion‐doped perovskite oxides is still lacking. Therefore, this review mainly introduces the elements and strategies of various common anions doped at O‐site of perovskite oxides, analyzes their influence on the physical and chemical properties of perovskites, and separately concludes their applications in electrocatalysis. This review will provide ideas and prospects for the development of subsequent anion doping strategies for high performance perovskite oxides.

## Graphical abstract

This review first describes the elements and strategies of various common anions doped at the O‐site of perovskite oxides, then discusses the influence on the physical and chemical properties of anion‐doped perovskite oxides, and presents the application of anion‐doped perovskite oxides in different fields of electrocatalysis. Finally, the challenges and perspectives of anion‐doped perovskite oxide catalysts are suggested.

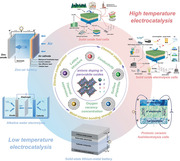

## 1 Introduction

**Figure 1:**
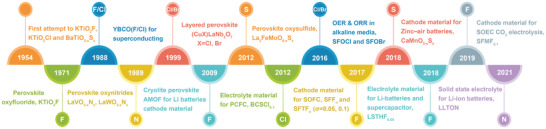
The chronological diagram of the development for anion doping in perovskite oxides.^[^ ^5^ ^−^ ^19^ ^]^ (YBCO(F/Cl) = Y_2_Ba_5_Cu_7_O_20−δ”_(F/Cl)_δ”'_, AMOF = Ag_3_MoO_3_F_3_, BCSCl_0.1_ = BaCe_0.8_Sm_0.2_O_2.9−δ_Cl_0.1_, SFOCl = Sr_2_FeO_2_Cl_2_, SFOBr = Sr_2_FeO_3_Br, SFF_σ_ = SrFeO_3−σ−δ_, SFTF_σ_ = SrFe_0.9_Ti_0.1_O_3−σ−δ_, LSTHF_0.05_ = Li_0.38_Sr_0.44_Ta_0.7_Hf_0.3_O_2.95_F_0.05_, SFMF_0.1_ = Sr_2_Fe_1.5_Mo_0.5_O_6−δ_F_0.1_, LLTON = Li_0.33_La_0.557_TiO_3−x_N_x_).

Perovskite oxide, a common type of oxide with a molecular formula of ABO_3_, was first discovered in perovskite ore as the calcium titanate (CaTiO_3_) compound.^[^ ^1^ ^]^ The cubic structure of perovskite ABO_3_ consists of [BO_6_] octahedra formed by B‐site ions and O ions, with eight octahedrons located at the vertices of the cube through a common angle. The A‐site ions are positioned at the center of the cube. The coordination numbers of the A‐site and B‐site are 12 and 6, respectively, in this structure. The A‐site element typically originates from the Ln rare earth, alkaline earth metal, or alkali metal groups, possessing a large radius and relatively low valence. Conversely, the B‐site element is typically composed of a transition metal element with a small radius and higher valence. According to the conclusion of Anthony F. et al.^[^ ^2^ ^]^ the tolerance factor (t) can be used to estimate the stability of the perovskite structure:

(1) $$t=\frac{r_{A}+r_{O}}{\sqrt{2}\left(r_{B}+r_{O}\right)}$$

where r_A_, r_B_, and r_O_ represent ionic radii of A‐site, B‐site, and O‐site ions, respectively. Perovskite oxide possesses a stable structure at the range of 0.75 < *t* < 1 and the lattice distorts to some extent with *t* deviation.^[^ ^3^ ^]^ Additionally, a series of perovskite derivatives exist, comprising of simple perovskite, double perovskite, triple perovskite, quadruple perovskite, and R‐P (Ruddlesden‐Popper) structure perovskite, which further expand the diversity of the perovskite family. The remarkable flexibility of perovskite oxides' elemental combination enables a vast number of periodic table elements to be doped into the A and B sites,^[^ ^4^ ^]^ which allows for the creation of solid solutions doped with one or more elements to meet various needs. In recent years, another doping route as the anion O‐site doping of perovskite oxides has attracted more and more research interests. Due to the occupation of oxygen sites by the doped anions, the regulation of oxygen vacancies by anion doping is more flexible compared to metal ion doping, and thus anion doping will be more effective than metal ion doping in the field of oxygen related electrochemical reactions. Anion doping enters oxygen sites by replacing lattice oxygen or occupying oxygen vacancies, using valence balance, electronegativity, and energy band structure to regulate the performance of perovskite oxides. For example, F‐doping will induce the formation of oxygen vacancies due to its higher electronegativity and lower negative charge, while S‐doping will enhance oxygen electrocatalytic activity by adjusting the electronic structure of the B‐site transition metal elements. The doping of anions at the O‐site typically represents only 0.1–0.2 of the O‐site stoichiometric ratio, but it can effectively regulate various properties of perovskite oxides, including their electronic and crystal structures, ion electronic conductivity, chemical stability, oxygen vacancy concentration and oxygen ion mobility. As a result, anion doping at the O‐site is widely considered a promising modification method for perovskite oxides, in addition to cation doping at the A/B‐site, which has gained increasing attention from researchers in recent years. A chronological diagram showcasing anion doping is displayed in **Figure** **1**. Perovskite oxide materials, employed as electrocatalysts, offer the benefits of both affordability and high efficiency. They possess extensive applications in low‐temperature electrocatalysis such as Li/Na‐ion batteries, Li/Na‐sulfur batteries, metal‐air batteries, supercapacitors, and high‐temperature electrocatalysis such as solid oxide fuel cells (SOFCs) and solid oxide electrolysis cells (SOECs), which have obtained significant attention.

Despite some existing reviews on the application of perovskite oxide materials in electrocatalysis, the majority of them have primarily focused on A/B‐site ion doping, neglecting the potential benefits of anion doping. As a result, a systematic summary of the application of anion doping in perovskite oxide materials is still lacking. Therefore, it is essential to summarize the current state of development, identify key challenges, and broaden the prospects for its future development.

**Figure 2:**
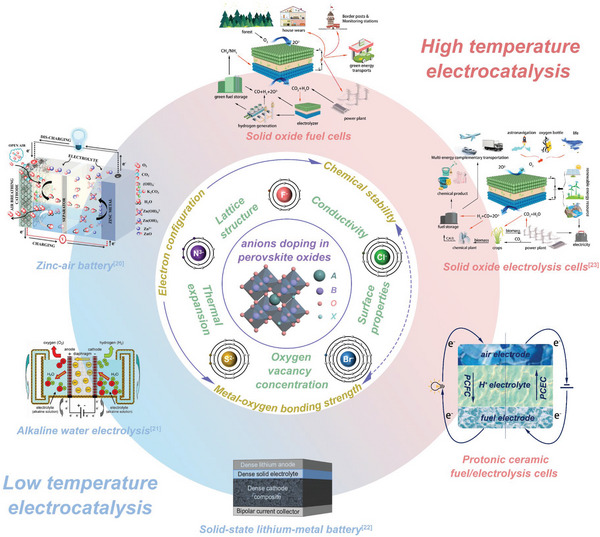
Physical and chemical properties and application examples of anion‐doped perovskite oxides. Reproduced with permission.^[^ ^20^ ^]^ Copyright 2014, American Chemical Society. Reproduced with permission.^[^ ^21^ ^]^ Copyright 2018, American Institute of Science. Reproduced with permission.^[^ ^22^ ^]^ Copyright 2018, Elsevier.

This review provides a comprehensive summary of the recent advances in anion O‐site doping of perovskites in the field of electrocatalysis. It focuses on the different methods and types of anion doping, corresponding characterization technologies, and the resulting effects on the physical and chemical properties of the materials. Additionally, the review analyzes the applications and stability of anion‐doped perovskites in electrocatalytic reactions, including but not limited to lithium batteries, zinc‐air batteries, and solid oxide cells (SOC), as illustrated in **Figure** **2**.

## 2 Anion Doping Types and Synthesis Processes

The anions including halogens (F, Cl, Br), S and N have been investigated in perovskite oxides O‐site doping in recent years. This section mainly concludes various anions doping and their related preparation processes in the past decade researches.

### 2.1 Halogens Doping

Halogen anions doping has been studied widely in perovskite oxides. The most common methods for synthesizing halogen doped perovskites are the sol‐gel self‐combustion synthesis method and the solid‐state reaction method.

#### 2.1.1 Sol‐Gel Self‐Combustion Method

The sol‐gel self‐combustion (SSC) method combines the advantages of the sol‐gel method and the self‐propagating high temperature synthesis (SHS) method, which is an energy‐efficient and environmentally friendly synthesis method.^[^ ^24^ ^]^ This method requires lower synthesis temperature and the inert gas (N_2_, CO_2_) generated by combustion can not only be used to prevent oxidation of the doped ions, but also avoid agglomeration of the synthesized powder particles to a certain extent. It occupies the advantages of low raw material cost, fast combustion reaction, high product purity, large specific surface area, and a porous structure, which make this method widely used in perovskites synthesis.

**Figure 3:**
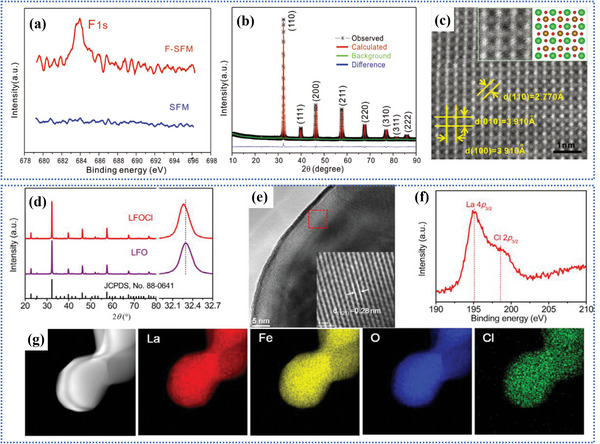
Structural characterizations for F‐SFM powders: a) X‐ray photoelectron spectroscopy (XPS) spectra F‐1*s* for SFM and F‐SFM. b) Refined room temperature X‐ray diffraction (XRD) profile. c) High‐resolution transmission electron microscopy (HRTEM) image of F‐SFM. Reproduced with permission.^[^ ^18^ ^]^ Copyright 2018, John Wiley and Sons. Structural characterizations for LFOCl powders: d) XRD profiles of LFO and LFOCl. e) HRTEM image of LFOCl. f) XPS spectra of La 4*p* and Cl 2*p* for LFOCl. g) high‐angle annular dark‐field scanning transmission electron microscopy (HAADF‐STEM) image and the corresponding elemental mapping images of LFOCl. Reproduced with permission.^[^ ^34^ ^]^ Copyright 2021, Elsevier.

Normally, for preparation of F‐ and Cl‐doping perovskite oxide through the typical SSC process, the soluble halide of the A‐site element serves as the halogen source.^[^ ^15^, ^18^, ^25^ ^−^ ^39^ ^]^ Li et al. used the SSC method to prepare F‐doped double perovskite Sr_2_Fe_1.5_Mo_0.5_O_6−δ_F_0.1_ (F‐SFM) as the cathode of SOEC for CO_2_ electrolysis.^[^ ^18^ ^]^ **Figure** **3**a–c demonstrate the existence of F element and the pure cubic phase. The preparation process for Cl‐doping is similar. Shao et al.^[^ ^34^ ^]^ designed a series of Cl‐doping perovskite LaFeO_3−x−δ_Cl_x_ (LFOCl, x = 0, 0.1) to improve the oxygen evolution reaction (OER) activity of perovskite LaFeO_3_. Figure 3d–g confirm the presence of Cl element, which is uniformly distributed in the LaFeO_3−x−δ_Cl_x_ particles, causing a certain degree of lattice expansion. Xiong et al.^[^ ^40^ ^]^ designed high active oxygen sites on Ba_0.5_Sr_0.5_Co_0.8_Fe_0.2_O_3−δ_ to improve its OER performance, using NH_4_F as the F source and hydrothermal treatment as the fluorinated process, which could also obtain F‐doped perovskite. Additionally, NH_4_F is also used for F‐doping by vapor fluorination. LaCoO_3_ was prepared by SSC method and placed in the lower air outlet, and NH_4_F was placed in the upper air outlet at a temperature of 400 °C in a tube furnace for 2 h, and then a series of F_x_‐LaCoO_3_ (x = 0, 0.05, 0.1, 0.2, and 0.3) was obtained.^[^ ^41^ ^]^ However, this fluorination process requires to be treated in Ar/N_2_ atmosphere. The common halogen sources and corresponding sintering temperature of SSC method are summarized in **Table** **1**. It's worth noting that for halogen doping, the sintering temperature is typically the same as that of the substrate, or 100 °C higher, and requires the same amount of holding time. Furthermore, due to the relatively low melting point of metal bromide (generally below 800 °C), it tends to volatilize during combustion and sintering, and as a result, there is no reported research on the synthesis of Br‐doping perovskite oxides using the SSC method.

**Table 1:** Summary of common halogen sources of SSC method.

Target products	Halogen sources	Sintering temperature and time	Refs
SrFeO_3−σ−δ_F_σ_ (σ = 0, 0.05, and 0.10)	SrF_2_	900 °C for 2 h	[15]
SrFe_0.9_Ti_0.1_O_3−σ−δ_F_σ_ (σ = 0, 0.05, and 0.10)	SrF_2_	1000 °C for 5 h	[15]
Sr_2_Fe_1.5_Mo_0.5_O_6−δ_F_0.1_	SrF_2_	1000 °C for 5 h	[18]
Sr_2_Fe_1.5_Mo_0.5_O_6−x−δ_F_x_ (x = 0, 0.1, 0.2 and 0.3)	SrF_2_	1100 °C for 5 h	[33]
BaCe_0.8_Sm_0.2_O_2.9−δ_F_0.1_	BaF_2_	1100 °C for 2 h	[25]
PrBaCo_2_O_5+δ_F_x_ (x = 0, 0.1 and 0.2)	BaF_2_	1100 °C for 3 h	[26]
Ba_0.5_Sr_0.5_Co_0.8_Fe_0.2_O_2.9−δ_F_0.1_	BaF_2_	1000 °C for 2 h	[28]
Ba_0.95_Ca_0.05_Fe_0.85_Sn_0.05_Y_0.1_O_2.9−δ_F_0.1_	BaF_2_	1000 °C for 3 h	[29]
BaCo_0.4_Fe_0.4_Zr_0.1_Y_0.1_O_2.95−δ_F_0.05_	NH_4_F	980 °C	[31]
Pr_2_NiO_3.9+δ_F_0.1_	NH_4_F	1200 °C for 5 h	[35]
Ba_0.5_Sr_0.5_Co_0.8_Fe_0.2_O_3−δ_F_x_ (x = 0, 0.1, 0.2 and 0.3)	NH_4_F	900 °C for 2 h	[40]
La_0.6_Sr_0.4_Co_0.2_Fe_0.8_O_3−δ−x_F_x_ (x = 0, 0.05 and 0.1)	SrF_2_	800 °C for 4 h	[27]
La_0.9_Sr_0.8_Co_0.4_Mn_0.6_O_3.9−δ_F_0.1_	SrF_2_	1100 °C for 10 h	[30]
LaFeO_3−δ−x_Cl_x_ (x = 0, 0.05, 0.1and 0.2)	LaCl_3_	800 °C for 5 h	[34]
LaBa_0.5_Sr_0.5_Fe_2_O_5.875−δ_F_0.125_	SrF_2_	1000 °C for 12 h	[36]
La_0.6_Sr_0.4_Fe_0.8_Ni_0.2_O_2.9−δ_F_0.1_	SrF_2_	950 °C for 5 h	[37]
La_0.5_Sr_0.5_FeO_3−x−δ_F_x_ (x = 0∼0.3)	SrF_2_	1000 °C for 5 h	[38]
La_0.5_Ba_0.5_FeO_3−x−δ_F_x_ (x = 0∼0.1)	LaF_3_	950 °C for 4 h	[39]

#### 2.1.2 Solid‐State Reaction Method

The solid‐state reaction method (SSR) is one of the most commonly used methods for the synthesis of perovskite oxides. The preparation process of SSR method is simpler than the SSC method, but requires a higher temperature. Specifically, solvents (ethanol, isopropanol, etc.), sintering aids and the raw powders with stoichiometry are fully mixed and ground. After drying treatment, the sample is sintered at high temperature to form the final product.^[^ ^42^ ^]^ Due to poor complexing ability of the alkali metals, there are hardly any research reports on the preparation of Na^+^/Li^+^‐containing perovskite materials by the SSC method, while the SSR method is often used to prepare Na^+^/Li^+^‐containing perovskite. Sun et al. provided synthesis and characterization of the solid−state electrolyte for Li‐ion battery as LiSr_1−0.5x_TiTaO_6−x_F_x_, which was obtained from SrCO_3_, Li_2_CO_3_, TiO_2_, LiF, and Ta_2_O_5_.^[^ ^43^ ^]^ Here, an excess of 10% Li_2_CO_3_ was used to address the issue of Li evaporation during high‐temperature heat processing. The thoroughly mixed starting materials were pre‐calcined at 800 °C for 2 h and then at 1100 °C for 10 h. Finally, the obtained powders were pressed at 20 MPa into pellets and sintered at 1250–1300 °C for 6 h. In addition, the SSR method can serve as an alternative approach to prepare perovskite oxides when the SSC method produces insoluble components, like metal halides CaF_2_.

It is worth noting that the halides of A‐site elements exhibit superior thermal, solubility, and chemical stability compared to those of B‐site elements. This could potentially explain why halides of A‐site elements are viable as halogen sources in both SSC and SSR methods. However, if the halides of B‐site elements display adequate thermal stability, solubility, and chemical stability that align with the preparation requirements, they can also theoretically be utilized as a halogen source.

Furthermore, it is also noteworthy that if the sintering temperature surpasses the boiling point of the bromide, the doping amount will deviate significantly from the anticipated value. Therefore, it is typically essential to combine the SSR method with dry pressing during Br‐doping to control the volatilization of Br. Luo et al. prepared Br‐doped BaCe_0.8_Gd_0.2_O_2.8±δ_Br_0.2_ (BCGBr) by the SSR method and sintered at 1250 °C for 5 h to obtain BCGBr powders.^[^ ^44^ ^]^ The proton conductor electrolyte was obtained after the powders were pressed at 250–300 MPa and sintered at 1600 °C for 10 h with 1 wt.% CuO as sintering additive. The common halogen sources and the sintering temperature utilized in SSR method are concluded in **Table** **2**. Additionally, it should be emphasized that Br^−^ is susceptible to oxidation by certain metal cations, such as Fe^3+^. The poor chemical and thermal stability of Br^−^ severely limits the synthesis of perovskite oxybromides.

**Table 2:** Summary of common halogen sources of SSR method.

Target products	Halogen sources	Sintering temperature and time	Refs
Ba_2_CaNbO_5.48_F_0.05_ Ba_2_CaNbO_5.48_Cl_0.05_	BaF_2_ BaCl_2_	900–1300 °C 800–1300 °C	[42]
LiSr_1−0.5x_TiTaO_6−x_F_x_	LiF	1300 °C for 6 h	[43]
BaCe_0.8_Gd_0.2_O_2.8±δ_Br_0.2_	BaBr_2_	1250 °C for 5 h	[44]
BaZr_0.8_Y_0.2_O_2.9−δ_F_0.1_	BaF_2_	1500 °C for 24 h	[45]
BaCe_0.9_Gd_0.1_O_2.9−δ_X_0.1_	BaX_2_ (X = F, Cl and Br)	1150 °C for 2 h	[46]
BaCe_0.8_Sm_0.2_O_2.9−δ_Cl_0.1_	BaCl_2_	1100 °C for 5 h	[13]
La_0.5_Ba_0.25_Sr_0.25_CoO_2.9−δ_F_0.1_	BaF_2_	1050 °C for 5 h	[47]
Nd_1.9_Ba_0.1_NiO_4+δ_F_γ_ (γ = 0, 0.03, 0.05, 0.07 and 0.1)	BaF_2_	1100 °C for 5 h	[48]
Sr_2_Co_1−x_Fe_x_O_3_Cl	SrCl_2_	850 °C for 24 h	[49]

### 2.2 N, S‐Doping

As for N and S doped perovskite oxides, due to their volatileness, the doping method cannot be as same as the previously mentioned halogen doping as sintering at high temperature after mixing the halogen source and raw materials of the substrate. Generally, the substrate needs to be synthesized first and then conducted nitriding or sulphurating treatment.

**Figure 4:**
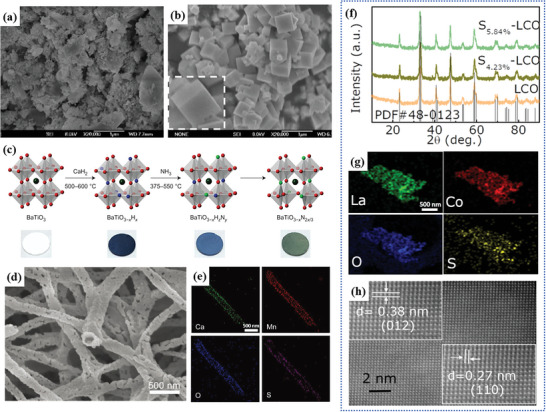
Comparison of the representative scanning electron microscopy (SEM) images of the NaTaO_3−x_N_x_ using a) the SSR method. Reproduced with permission.^[^ ^57^ ^]^ Copyright 2010, Elsevier and b) the hydrothermal method. Reproduced with permission.^[^ ^58^ ^]^ Copyright 2011, Elsevier. c) Two‐step synthesis of oxynitride BaTiO_3−x_N_2x/3_. Reproduced with permission.^[^ ^60^ ^]^ Copyright 2015, Springer Nature. d) SEM and e) Energy dispersive spectrometer (EDS) mapping of S‐doped CaMnO_3_ at 300 °C sulphuration. Reproduced with permission.^[^ ^17^ ^]^ Copyright 2018, John Wiley and Sons. f) XRD patterns of LCO and S‐LCO. g) EDS mapping and h) HRTEM image of S_5.84%_‐LCO. Reproduced with permission.^[^ ^61^ ^]^ Copyright 2020, American Chemical Society.

At present, methods of doping perovskite with N generally include thermal ammonolysis method, hydrothermal method, topological chemical method and so on. The common thermal ammonolysis method generally puts the substrates or precursors of perovskite oxides under a certain ammonia flow at certain temperatures.^[^ ^50^ ^−^ ^54^ ^]^ Depending on the perovskite variety and the doping amount, the required temperature and flow are different. Kim et al.^[^ ^50^ ^]^ synthesized N‐doped SrMn_0.2_M_0.8_O_2.6_N_0.4_ (M = Nb, Ta) using the uniformly mixed Sr_5_M_4_O_15_ and MnCl_2_ dry‐pressed pellets as the precursors and heated them in a 100 sccm NH_3_ flow for 12 h at the specified temperature. In another work, Li_0.33_La_0.557_TiO_3_ (LLTO) nanofibers were prepared by electrospinning, and then loaded under 100 sccm NH_3_ flow at 525–600 °C for 2 h respectively to control the nitrogen doping according to the temperature.^[^ ^19^ ^]^ The thermal ammonolysis method is the most common and also sometimes used in the synthesis of other N‐doped oxides,^[^ ^55^, ^56^ ^]^ but this method cannot be quantitative. Although it can be adjusted to a certain extent through the flow rate of NH_3_ and treatment temperature and time, this method is not a normal linear increase relationship, which still needs to be further verified by the subsequent N content detection, while the SSR method could control the amount of N doping by quantifying the nitrogen source. For instance, Jiang et al.^[^ ^57^ ^]^ found that N‐doped NaTaO_3_ compounds (NaTaO_3−x_N_x_) could be synthesized by SSR method using NaTaO_3_ prepared at low calcination temperature as starting material and melamine (C_3_H_6_N_6_) as nitrogen source. However, SSR method is sometimes difficult to control the homogeneity, crystallinity and particle size of the product. Further, Jiang et al. changed the synthesis process and switched to the hydrothermal method,^[^ ^58^, ^59^ ^]^ which is more homogeneous for components and more uniform in the particle size compared with the SSR method, as shown in **Figure** **4**a,b.

Despite the potential benefits of the hydrothermal method for anion doping, the low mobility of N^3−^ species in the target perovskite lattice can impose serious restrictions on the composition and structure, making it difficult to achieve successful anion doping. A novel topological chemical route was proposed to explore oxynitride photocatalysts by Yajima et al.,^[^ ^60^ ^]^ using the instability of H^−^ in the perovskite type hydroxide BaTiO_3−x_H_x_ (x≤0.6), and the ammonolysis can be triggered just at low‐temperature condition (375–550 °C). Then the exchange of H^−^ to N^3−^ took place through the mixed intermediate O−H−N, consequently BaTiO_3−x_N_2x/3_ was obtained, the total route is exhibited in Figure 4c. The use of the “labile hydride” strategy shows promising in the exploration of different oxynitrides and potentially other anions, such as Br^−^, in perovskite materials.

The introduction of S into the perovskite lattice usually be realized by SSR method^[^ ^62^ ^]^ or hydrothermal reaction.^[^ ^63^ ^]^ Jiang et al. used the hydrothermal system to synthesize S‐doped NaTaO_3_ using Na_2_S_2_O_3_ as sulfur source.^[^ ^63^ ^]^ Another novel thermal sulfur vapor method for preparing S‐doping perovskites was reported by Peng et al.^[^ ^17^ ^]^ The prepared CaMnO_3_ nanofibers were treated in inert protective atmosphere Ar or N_2_, then the starting materials were placed in the lower air outlet at a temperature of 200 to 400 °C in a tube furnace, and the S powders were sintered at the upper air outlet. The SEM image and EDS mapping of the sulphurated CaMnO_3_ nanofibers are shown in Figure 4d,e. S element is evenly distributed in the nanofibers by this method. Ran et al.^[^ ^61^ ^]^ further confirmed the feasibility of this method in synthesis of S‐doped LaCoO_3_, as shown in Figure 4f. No impurity peaks were detected in the XRD profile. Figure 4g demonstrated that S was rather uniformly distributed and the interplanar distances of 0.38 and 0.27 nm in Figure 4h were consistent with their (012) and (110) planes, respectively. Sulphuration reaction using S vapor was a kinetic‐driven process like the thermal ammonolysis method for N‐doping. Therefore, S will preferentially enter the perovskite surface lattice rather than diffuse into the inner lattice.

**Table** **3** summarizes the synthetic methods discussed above. Currently, doping processes involving F and Cl for perovskite oxides primarily use the SSC and SSR methods, which are well‐established. As for powder morphology, the SSC method typically produces finer particles compared to the SSR method. Nevertheless, researches on the adjustment of morphology are scarce, possibly due to the high sintering temperature requirements of substrates, which make the commonly used hydrothermal method challenging to implement. Despite being widely used in the synthesis of perovskite oxide materials with nanofibrous structures in recent years, there is no related research report on F, Cl‐doping of electrospinning, which presents an interesting research direction worth exploring. Furthermore, the thermal method commonly used for N, S‐doping presents challenges in accurately synthesizing the expected doping amount, and only allows for passive detection of the synthesized doping amount during the experiment. The topological chemistry method, developed by Yajima et al.,^[^ ^60^ ^]^ holds great promise in resolving this dilemma by quantifying unstable intermediates prior to doping. Furthermore, while perovskite bromides have garnered significant attention in recent years, the synthesis of bromine‐doped perovskite oxides remains largely unexplored, except for the SSR method. Considering the success of N, S‐doping methods, as well as topological chemical method, these alternative approaches may be worth investigating.

**Table 3:** Summary of anion doping types and synthesis processes.

Methods	Application	Advantages	Disadvantages
Sol‐gel self‐combustion	F, Cl‐doping	Homogeneous High purity Large specific surface area	Low yield
Solid state reaction	All anions doping	Large yield Widely applied	Heterogeneous Higher temperature required
Thermal ammonolysis	N‐doping	Simple process	Difficult to quantify
Hydrothermal reaction	N, S‐doping	Capable to adjust the morphology	Smaller doping amount
Topological chemical	N‐doping	Capable to quantify Promising for other anions doping	High cost
Thermal sulfur vapor	S‐doping	Simple process	Difficult to quantify

## 3 Physical and Chemical Properties

Before delving into the effects of anion doping on the properties of perovskite, it is crucial to understand how anions enter the perovskite lattice. As depicted in Figure 1, there are two distinct routes that anions can be doped into the perovskite lattice. The first involves anions replacing oxygen ions within the crystal lattice or occupying oxygen vacancies, while the second involves anions occupying interlayer gaps in the crystal structure. Specifically, for single perovskites, only F doping into the interlayer gap is feasible because other anions have larger radii than oxygen ions, and their entry into the interlayer gap may cause significant lattice distortion. However, for double perovskites or layer perovskites, the aforementioned anion ingresses are achievable owing to their substantial interlayer gaps. The presence of anions in different locations within the lattice can significantly impact the physical‐chemical properties and catalytic activity of the perovskite oxides.

### 3.1 Physical Properties

Regarding the influence on physical properties, anion doping mainly affects the crystal structure, thermal expansion, electrical conductivity, oxygen vacancy concentration and surface properties of perovskite oxides. Specifically, the ionic radius, band structure, and valence state of the B‐site transition metal ions are the basic factors to be considered.

#### 3.1.1 Lattice Structure

**Figure 5:**
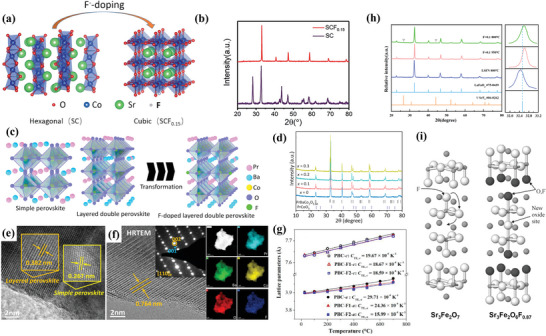
a) Schematic presentation of the structure transition from hexagonal structure to cubic structure. b) XRD patterns of as‐prepared SCF_0.15_ and SrCoO_3−δ_ (SC) samples. Reproduced with permission.^[^ ^65^ ^]^ Copyright 2019, Royal Society of Chemistry. c) Schematic illustration of phase transformation after F‐doping. d) Room‐temperature XRD patterns of the P_1.1_BCOF_x_ (x = 0–0.3) samples. e) HRTEM image of the Pr_1.1_Ba_0.9_Co_2_O_5+δ_ sample f) HRTEM and HAADF‐STEM images of the P_1.1_BCOF_0.1_ sample. Reproduced with permission.^[^ ^66^ ^]^ Copyright 2023, Elsevier. g) PBC‐Fx thermal expansion curves along with lattice parameters a and c. Reproduced with permission.^[^ ^26^ ^]^ Copyright 2018, Elsevier. h) XRD patterns of LSFNF_0.1_ at different sintered temperatures and LSFN. Reproduced with permission.^[^ ^37^ ^]^ Copyright 2022, American Chemical Society. i) Structures of Sr_3_Fe_2_O_7_ and Sr_3_Fe_2_O_6_F_0.87_. Oxide ions are shown in white, the mixed oxide/fluoride site in dark grey. Reproduced with permission.^[^ ^67^ ^]^ Copyright 1999, Royal Society of Chemistry.

The lattice is a spatial framework characterized by its inherent regularity. When anions are introduced into the perovskite lattice, it can cause alterations in the structural regularity and stability of the crystal. In the case of La_1−x_Sr_x_MnO_3_, high fluorination has been demonstrated to reduce its structural stability.^[^ ^64^ ^]^ The lattice parameters and unit cell volume of the perovskite decreased with the increase of the F‐doping amount. Moreover, when La_1−x_Sr_x_MnO_3−2x+δ_F_2x_ was undergone a structural transformation from rhombic to orthorhombic crystal at x = 0.30. At x>0.33, impurities such as SrMnO_3_ and even La_2_O_3_ were generated, and a phase change occurred in the samples, indicating a decrease in structural stability. However, during the research process, the ratio of La and Sr also changed with the doping amount of F, and the effect of fluorination on lattice stability needs to be further verified. Besides, proper fluorination is capable to convert the crystal structure into a more stable configuration. Researches have indicated that the hexagonal phase of Sr_2_Co_2_O_5_ can be transformed into the cubic phase of SrCoO_2.85−δ_F_0.15_ (SCF_0.15_) after F‐doping with the CoO_6_ octahedron undergoing a change from coplanar to co−angular, as illustrated in **Figure** **5**a,b.^[^ ^65^ ^]^ Zhao et al. have further confirmed this effect in the investigation of the F‐doped Pr_1.1_Ba_0.9_Co_2_O_5+δ_F_0.1_ (P_1.1_BCOF_0.1_) that introducing the F^−^ anions into the lattice may induce the cation ordering of Pr^3+^ and Ba^2+^, which makes the Pr_1.1_Ba_0.9_Co_2_O_5+δ_ transform from cubic/tetragonal to tetragonal symmetry, as demonstrated in Figure 5c–f.^[^ ^66^ ^]^

F‐doping also has a significant impact on the symmetry of the crystal. Specifically, in so far as Sr_2_Fe_1.5_Mo_0.5_O_6−x−δ_F_x_ (SFMF), F‐doping resulted in a transformation of the crystal structure from an orthogonal configuration (with space group *Cmmm*) to a pseudo cubic configuration (with space group *Pm*3—*m*), which enhanced the lattice symmetry of SFMF.^[^ ^33^ ^]^ In addition, in Figure 5i, research has demonstrated that partially replacing the O atoms in the perovskite Sr_3_Fe_2_O_7_ lattice with F would also lead to the expansion of the crystal lattice along the c‐axis.^[^ ^67^ ^]^ Moreover, the R‐P perovskite series Ln_1.2_Sr_1.8_Mn_2_O_7_F_2_ (Ln = La, Pr, Nd, Sm, Eu, Gd) showed a significant expansion in the distance between perovskite layers along the crystal c‐axis after F‐doping, while a larger contraction was observed along the a‐b plane.^[^ ^68^ ^]^

Different from introducing into the layer gap, when F doping into the lattice site, it will lead to lattice contraction, due to its smaller ionic radius than O^2−^. As shown in Figure 5h, during the investigation of the synthesis of F‐doped La_0.6_Sr_0.4_Fe_0.8_Ni_0.2_O_3−δ_, it was found that at a sintering temperature of 800 °C,^[^ ^37^ ^]^ F was unable to fully incorporate into the lattice structure, with an impurity phase of SrF_2_ existed, and finally the pure phase La_0.6_Sr_0.4_Fe_0.8_Ni_0.2_O_2.9−δ_F_0.1_ (LSFNF_0.1_) was obtained at 950 °C. As the degree of fluorination increased, the main peak in the XRD pattern of LSFNF_0.1_ shifted towards the right, indicating a gradual contraction of the crystal lattice.^[^ ^37^ ^]^ In contrast, doping with elements such as Cl, Br, N and S, etc., as the ionic radii are larger than O^2−^, would cause the lattice expanding to a certain extent. In this case, lattice distortion would occur due to the change of the electronic structure.^[^ ^12^, ^46^, ^58^, ^63^ ^]^

It is worth noting that the majority of perovskite oxide crystal structures are categorized as ionic crystals, mainly resulting from the combination of metal and non‐metal elements via ionic bonds (partial covalent bonds). However, the structural model of perovskite is generally depicted in its atomic form, as displayed in Figure 5a. It should be pointed out that the ionic radius of O^2−^ is considerably larger than that of common B‐site metal elements (such as Fe, Co, Ni, and others), but in certain structure drawing software such as VESTA, the automatically generated ball‐and‐stick model uses rigid atoms, which makes it easy for some readers and researchers to ignore the actual ionic bonding form and has occurred in many studies.

Overall, the impact of anion doping on the lattice structure should mainly consider its ionic radius and the change of cation valence state caused by the change of its ion valence state, which affects the size of the cation radius, and then affects the tolerance factor (*t*) of the entire perovskite. The changes in the perovskite lattice structure discussed above are all based on the comprehensive reflection of these effects.

#### 3.1.2 Thermal Expansion

Cobalt‐based perovskites typically demonstrate exceptional performance in oxygen reduction reaction (ORR) owing to their high‐volume diffusion coefficient and oxygen surface exchange coefficient.^[^ ^69^ ^−^ ^73^ ^]^ In spite of this, at high temperatures, the spin state transition of Co ions can trigger chemical expansion, leading to a weakening of the electrode‐electrolyte interface contact. Therefore, the application of cobalt‐based perovskite as PrBaCo_2_O_5+δ_ (PBC) is limited by its high coefficient of thermal expansion (CTE) comparing with GDC (α = 12.5 × 10^−6^ K^−1^).^[^ ^74^, ^75^ ^]^ It is obvious in Figure 5g that F‐doping mainly reduces CTE in the a‐b plane, while makes little difference along the c‐axis. As a tetragonal double perovskite, PBC predominantly features oxygen vacancies in the a‐b plane, which is consistent with its higher CTE along the a‐ and b‐axis than along the c‐axis. The variation in CTE along the a, b, and c direction is closely linked to the distribution of oxygen vacancy concentration.^[^ ^26^ ^]^ The anisotropy of oxygen vacancy concentration distribution could be inferred based on the anisotropy of the CTE, which is helpful to know oxygen ion diffusion rate, route and explore the mechanism of the effect of F‐doping on the thermal expansion in the perovskite. The thermal expansion behavior of perovskite oxides (such as PBC) could be mainly attributed to chemical expansion resulting from reduction of B‐site transition metal ions (Co^n+^) and/or their low/high spin transition.^[^ ^76^ ^]^ Furthermore, due to higher electronegativity of fluorine compared to that of oxygen, it becomes more arduous for Co^n+^ to acquire electrons upon F‐doping, which consequently hinders the reduction of Co^n+^ and suppresses the formation of oxygen vacancies and meanwhile the CTE along the direction is reduced.

The impact of anion doping on the thermal expansion properties of materials stems primarily from its direct influence on the valence state of B‐site transition metal ions and its indirect influence on the concentration of oxygen vacancy. While research on the effects of anion doping other than fluorine is limited, it can be anticipated that such effects are closely tied to the concentration of oxygen vacancy.

#### 3.1.3 Electrical Conductivity

In the electrocatalytic process, effective electron flowing through the electrodes (electronic conductivity) is essential for generating high current. Additionally, in battery/cell systems, the electrolyte must possess high ionic conductivity to accelerate charge transfer rates and promote high‐efficiency catalysis, ultimately improving electrochemical efficiency.

**Figure 6:**
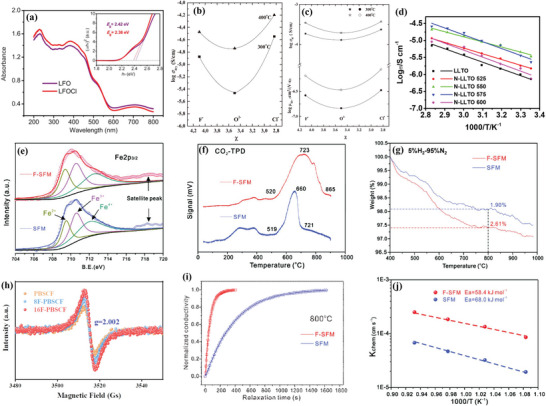
a) UV–vis absorption spectra of LFO and LFOCl. Inset is the corresponding Tauc plots. Reproduced with permission.^[^ ^34^ ^]^ Copyright 2021, Elsevier. b) Conductivity at dry air condition versus electronegativity at 300 and 400 °C for the compositions Ba_2_CaNbO_5.5_, Ba_2_CaNbO_5.48_F_0.05_ and Ba_2_CaNbO_5.48_Cl_0.05_. c) Proton mobility $\mu_{H^{+}}$ and proton conductivity $\sigma_{H^{+}}$ versus electronegativity for the compositions Ba_2_CaNbO_5.5_, Ba_2_CaNbO_5.48_F_0.05_ and Ba_2_CaNbO_5.48_Cl_0.05_. Reproduced with permission.^[^ ^42^ ^]^ Copyright 2018, Elsevier. d) Total conductivity of LLTO and N‐doped LLTO treated in different temperature. Reproduced with permission.^[^ ^19^ ^]^ Copyright 2021, IOPscience. Physicochemical properties of F‐SFM and SFM: e) XPS Fe‐2p_3/2_ spectra. f) CO_2_‐TPD profiles. g) TG profiles in 5%H_2_‐95%N_2_. Reproduced with permission.^[^ ^18^ ^]^ Copyright 2018, John Wiley and Sons. h) EPR plots measured at room temperature. Reproduced with permission.^[^ ^79^ ^]^ Copyright 2022, Elsevier. i) Normalized conductivity relaxation profiles of the F‐SFM and SFM at 800 °C. j) Oxygen surface reaction rate constant *k*_chem_ of the F‐SFM and SFM versus 1000/T. Reproduced with permission.^[^ ^18^ ^]^ Copyright 2018, John Wiley and Sons.

Generally, the electron conductance predominantly involves the conduction mechanism of the small polarons, which exhibits two modes of movement within the lattice. The small polarons conduct through the energy band when the electron‐lattice coupling is low at lower temperature. In this context, the explanation for how halogen doping influences the electronic conductivity of perovskite oxides is concerned with calculating the corresponding optical band gap according to the Tauc equation, where halogen doping usually results in a narrower band gap for the perovskite substrate. For instance, the optical band gap of LaFeO_3−δ_ (LFO) could be reduced from 2.42 to 2.38 eV after Cl‐doping, which could reduce the energy required to excite charge carriers to the conduction band, as shown in **Figure** **6**a, resulting in a promotion in electronic conductivity.^[^ ^34^ ^]^ At higher temperatures, the situation becomes more complex as the electron−lattice coupling is intensified, resulting in the small polaron transforming into a localized charged particle and hopping through the lattice to conduct. Consequently, the impact of halogen ion doping on electronic conductivity centers on alterations in the small polaron concentration, B‐site metal ion valence state, and oxygen vacancy concentration. However, the outcome of halogen anion doping varies across different material systems. While it usually enhances electronic conductivity,^[^ ^26^, ^29^, ^37^ ^]^ it may also partially lead to a reduction.^[^ ^27^, ^30^, ^32^, ^48^ ^]^

The mechanism of anion doping for ionic conductivity relies on the mixed anion effects. In the research of F, Cl‐doped BaCaNbO_5.5_, Tarasova et al. discovered that despite the difference in dopant radii and electronegativity, both F and Cl doping resulted in an increase in oxygen conductivity (under dry air conditions), as illustrated in Figure 6b.^[^ ^42^ ^]^ Additionally, the proton mobility and proton conductivity depicted in Figure 6c also follow a similar trend. Based on the above results, the authors concluded that the improvement in ionic conductivity was not due to the change in electronic density but rather, the electrostatic repulsion between different anions (O^2−^ and X^−^) located on the same sublattice. In other words, changes in oxygen mobility caused by the repulsion between positively charged defects ($V_{O}^{\cdot \cdot }$ and $F_{O}^{\cdot }$/$Cl_{O}^{\cdot }$) facilitated the enhancement of the ionic conductivity of perovskite oxides.

Moreover, the investigation of N‐doped Li_0.33_La_0.557_TiO_3_ (LLTO) nanofibers with poly(vinylidene fluoride‐co‐hexafluoropropylene) (PVDF‐HFP) as a composite electrolyte material for solid‐state lithium batteries has revealed a total ionic conductivity of 4.28 × 10^−6^ S cm^−1^, which is nearly three times higher than that of the undoped material, as depicted in Figure 6d.^[^ ^19^ ^]^ This improvement can be attributed to the ability of N‐doping to accommodate larger lattice distortions and weaken the bonding strength of adjacent Li−O bonds, which reduces the barrier of Li^+^ hopping migration, thus facilitates hopping involving several of the higher‐energy states.

#### 3.1.4 Oxygen Vacancy Concentration

Among the various types of defects present in perovskite materials, oxygen vacancies stand out as the most prevalent, exerting a crucial impact on the electronic and crystal structures, as well as on the oxygen migration and surface adsorption properties of the system. In the field of electrocatalysis, oxygen vacancies of the catalyst play a dual role as they serve as active sites for the reaction substrate and represent the primary conduit for oxygen ion transportation.

The effect of anion doping on the oxygen vacancy concentration mainly relies on the route of doping. When halide ions substitute the lattice oxygen directly, for the sake of maintaining electric neutrality in the system, the oxidation state of nearby metal cations will consequently decrease, making the lattice oxygen more easily lost, and simultaneously increasing the oxygen vacancy concentration. For instance, in F‐doped Sr_2_Fe_1.5_Mo_0.5_O_6−δ_ (SFM), O^2−^ was replaced by F^−^, which resulted in the partial transfer of dominated Fe ions from Fe^4+^ to Fe^3+^, adjusting the average oxidation state from Fe^3.17+^ of SFM to Fe^3.06+^ of F‐SFM, as presented in Figure 6e.^[^ ^18^ ^]^ The specific process is shown as Equation (1). Equations (2) and (3) are respectively represent the processes under oxidizing and reducing atmospheres. The CO_2_‐TPD (temperature programmed desorption) and TG (thermogravimetric) analysis in Figure 6f,g were both utilized to further demonstrate the effect of F‐doping on the oxygen concentration.^[^ ^18^ ^]^ In addition, the increase of oxygen vacancy concentration by F‐doping into lattice sites has also been observed in many other literatures.^[^ ^35^, ^37^, ^38^ ^]^ As shown in Figure 6h, in terms of PrBa_0.5_Sr_0.5_Co_1.5_Fe_0.5_O_6−x−δ_F_x_ (x = 0, 0.08, 0.16, denoted as, PBSCF, 8F‐PBSCF, 16F‐PBSCF), the EPR (Electron paramagnetic resonance) peak at g = 2.002 corresponds to electrons in oxygen vacancies,^[^ ^77^, ^78^ ^]^ and its signal intensity follows the order of PBSCF < 8F‐PBSCF < 16F‐PBSCF, indicating that 16F‐PBSCF has the highest concentration of oxygen vacancies among the materials.^[^ ^79^ ^]^

(2) $${\mathrm{SrF}}_{2}+O_{O}^{\times }+2{\mathrm{Fe}}_{\mathrm{Fe}}^{•}\overset{\mathrm{SrO}}{\to }{\mathrm{Sr}}_{\mathrm{Sr}}^{\times }+2F_{O}^{•}+2{\mathrm{Fe}}_{\mathrm{Fe}}^{\times }+O_{2}$$

(3) $$O_{O}^{\times }+2{\mathrm{Fe}}_{\mathrm{Fe}}^{•}\to 2{\mathrm{Fe}}_{\mathrm{Fe}}^{\times }+\frac{1}{2}O_{2}+V_{O}^{••}$$

(4) $$O_{O}^{\times }+2{\mathrm{Fe}}_{\mathrm{Fe}}^{\times }\to 2{\mathrm{Fe}}_{\mathrm{Fe}}^{\prime }+\frac{1}{2}O_{2}+V_{O}^{••}$$

When anions substitute oxygen vacancies or interlayer gaps in the crystal structure, the resulting decrease in concentration is evident if oxygen vacancies are replaced, and the introduction of anions in interlayer gaps also follows the principle of electrical neutrality, causing an increase in the oxidation state of adjacent cations. On the contrary, in such cases, lattice oxygen becomes less likely to be lost, leading to an overall decrease in oxygen vacancies concentration. Regarding F‐doped Ba_0.5_Sr_0.5_Co_0.8_Fe_0.2_O_3−δ_, F^−^ occupation of oxygen vacancies and interlayer gaps can be expressed by Equations (5) and (6), respectively.^[^ ^28^ ^]^

(5) $${\mathrm{BaF}}_{2}+V_{o}^{••}\overset{\mathrm{BaO}}{\to }{\mathrm{Ba}}_{\mathrm{Ba}}^{\times }+2F_{o}^{•}$$

(6) $${\mathrm{BaF}}_{2}\overset{\mathrm{BaO}}{\to }{\mathrm{Ba}}_{\mathrm{Ba}}^{\times }+F_{o}^{•}+F_{i}^{\prime }$$

#### 3.1.5 Surface Properties

Surface properties, such as surface oxygen exchange activity and specific area, can provide insights into the performance of many ionic‐electronic mixed perovskite materials, which is strongly dependent on the rapid oxygen exchange at the relevant gas/solid interface. This exchange process may ultimately dictate the overall catalytic reaction kinetics.^[^ ^80^ ^]^ Given that heterogeneous catalysis predominantly occurs on the surface of the catalyst, the catalytic performance is closely linked to the surface‐active sites and the specific surface area.

Recent experimental investigations indicate that anion doping can enhance the surface oxygen exchange activity while makes little negative change to the specific area. For instance, Li et al. compared the surface oxygen exchange coefficient (*k*_chem_) of SFM and F‐SFM by electrical conductivity relaxation (ECR) method,^[^ ^18^ ^]^ as shown in Figure 6i. After a sudden change of the surrounding atmosphere from 2:1 CO‐CO_2_ to 1:1 CO‐CO_2_ at 800°C, it took about 1000 s for the conductivity to return to equilibrium for F‐SFM while SFM took 4300 s, indicating that F‐SFM had a higher *k*_chem_. Figure 6j illustrated the temperature dependence of the *k*_chem_ for F‐SFM and SFM. It could be found that F‐SFM showed less dependence due to its lower activation energy. The *k*_chem_ at 700 °C of F‐SFM was 13.50 × 10^−5^ cm s^−1^, which was more than four times higher as 3.24 × 10^−5^ cm s^−1^ for SFM.^[^ ^18^ ^]^ This result was confirmed in another F‐doped SFM work.^[^ ^33^ ^]^ Similarly, SrFeO_3−σ−δ_F_σ_, SrFe_0.9_Ti_0.1_O_3−σ−δ_F_σ_ (σ = 0.05 and 0.1),^[^ ^15^ ^]^ SrCo_0.9_Nb_0.1_O_3−δ_F_0.1_,^[^ ^81^ ^]^ La_0.5_Ba_0.5_FeO_2.9−δ_F_0.1_^[^ ^39^ ^]^ and many other perovskite oxides with F‐doping all showed higher surface oxygen exchange activity than the undoped ones.^[^ ^26^, ^28^, ^31^, ^79^, ^82^ ^]^ However, all the investigations mentioned above have focused exclusively on F‐doping, while researches about other anions doping in relation to this aspect have not been reported yet. Moreover, the improved performance in surface activity is essentially due to the modification after anion doping in the electronic structure of the perovskite oxides.

As to the specific surface area, it is generally measured by the Brunauer‐Emmett‐Teller (BET) method. Anion doping will lower the surface area to some extent. The surface area of La_1.85_Sr_0.15_CuO_4_ *_+_*_δ_ decreased from 2.04 to 1.92 m^2^ g^−1^ and 1.97 m^2^ g^−1^ after F^−^ and Cl^−^ partially substituting O^2−^. Nd_1.85_Ce_0.15_CuO_4+δ_ also exhibited similar result.^[^ ^83^ ^]^ Furthermore, La_x_Sr_1−x_FeO_3−δ_Cl_σ_ series perovskite oxides possess smaller surface area than the parent oxides.^[^ ^84^ ^]^ Nevertheless, Zhu et al. found that as the amount of Cl‐doping increased, the surface area of LaFeO_3−δ−x_Cl_x_ (x = 0.05, 0.1 and 0.2) increased slightly.^[^ ^34^ ^]^ In fact, specific surface area is mainly dependent on the preparation process (For example, in the SSC method, the glycine method produces finer powders than the citric acid method), while the effect of anion doping is minimal.

### 3.2 Chemical Properties

Anion doping also shows significant effect on chemical properties, such as electronic configuration, chemical stability, and metal‐oxygen bonding strength. Specifically, molecular orbitals, chemical basicity, and electronegativity are the key factors in this process. By considering these factors in a comprehensive way, a better understanding and explanation of the mechanism of anion doping effect on chemical properties can be achieved.

#### 3.2.1 Electronic Configuration

**Figure 7:**
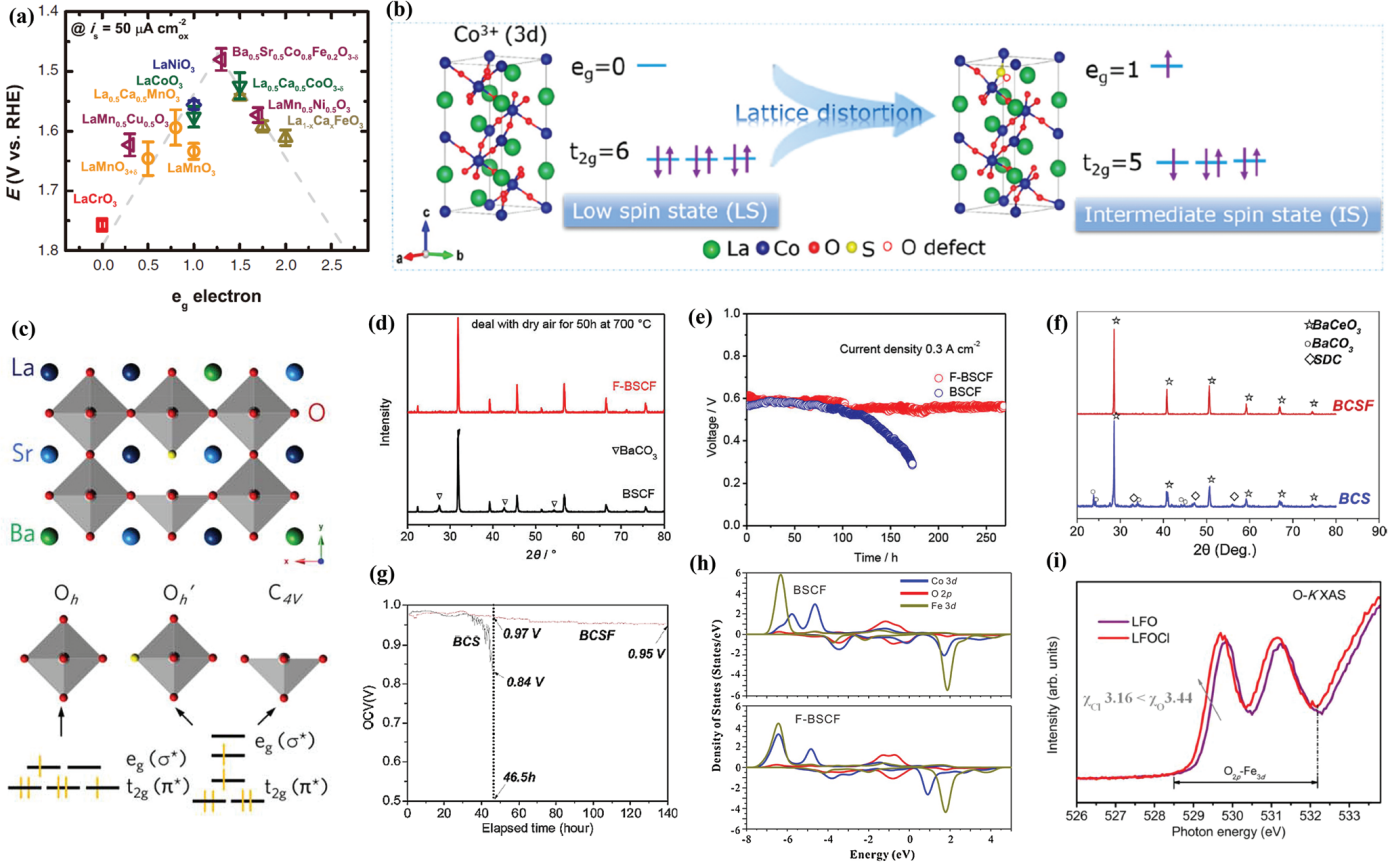
a) The relationship between the catalytic activity of OER and the number of e_g_ electrons possessed by the B‐site metal cation. Reproduced with permission.^[^ ^85^ ^]^ Copyright 2011, AAAS. b) Schematic diagram of intermediate spin state evolution and electron transition from t_2g_ to e_g_ orbital. Reproduced with permission.^[^ ^61^ ^]^ Copyright 2020, American Chemical Society. c) Schematic image of the perovskite structure and featured CoO_6−δ_ polyhedron. Reproduced with permission.^[^ ^86^ ^]^ Copyright 2018, Elsevier. d) XRD spectra of F‐BSCF and BSCF after treatment at 700 °C in streaming dry air for 50 h. e) Comparison between F‐BSCF and BSCF for long‐term stability, measured at 700 °C. Reproduced with permission.^[^ ^28^ ^]^ Copyright 2018, John Wiley and Sons. f) XRD spectra of BCS and BCSF pellets after treatment in the flowing dry air at 700 °C for 72 h. g) Long‐term characterization of single cells with BCS and BCSF electrolytes measured at 700 °C. Reproduced with permission.^[^ ^25^ ^]^ Copyright 2015, Elsevier. h) Projected density of states (PDOS) of Co 3*d*, O 2*p* and Fe 3*d* orbits in BSCF and F‐BSCF. Reproduced with permission.^[^ ^40^ ^]^ Copyright 2019, Elsevier. i) The O‐K soft X‐ray absorption spectroscopy (sXAS) spectra LFO and LFOCl. Reproduced with permission.^[^ ^34^ ^]^ Copyright 2021, Elsevier.

Anion doping will also affect the electronic configuration of perovskites. Goodenough et al.^[^ ^85^ ^]^ comprehensively investigated the influence of the 3*d‐*e_g_ orbital electron number of B‐site cations on the OER/ORR performance, as shown in **Figure** **7**a. For this series of perovskites, when the actual electron occupancy of the e_g_ orbital range is 1 to 1.5, its electronic structure can generate the most ideal OER/ORR catalytic activity. Take into account this consideration, S was doped into LaCoO_3_ in order to adjust its electron configuration and increase the spin state of Co^3^ *^+^*, thereby enhancing the OER performance of LaCoO_3_.^[^ ^61^ ^]^ As shown in Figure 7b, after doped with S, the lattice of LaCoO_3_ was distorted, and Co^3^ *^+^* changed from a low spin state to an intermediate spin state. Doping S^2−^ into CaMnO_3_ also has a similar effect. Through density functional theory (DFT) calculation, Peng et al. found that S‐doping would shorten the band gap of CaMnO_3_ and improve the electronic feedback ability of the active center Mn, thus enhancing the adsorption capacity of O_2_ during ORR.^[^ ^17^ ^]^ In a separate study, Luo et al. reported a perovskite oxyfluoride catalyst, La_0.5_Ba_0.25_Sr_0.25_CoO_2.9−δ_F_0.1_ (LBSCOF).^[^ ^86^ ^]^ F‐doping induced the presence of multiple, distinct Co and O sites, forming a square pyramidal symmetry (C_4V_) and an extra type of octahedral symmetry (O_h_′), as visually demonstrated in Figure 7c. Each of them possessed distinct orbital symmetries and doping dependencies, resulting in a complex reconfiguration of the molecular orbitals in the e_g_ (σ*) and t_2g_ (π*) orbitals.

The current research on the electronic configuration of other anions doping is lackluster, while it is evident that studying the electronic configuration can provide theoretically innovative perspectives for designing perovskite oxides at an atomic level.

#### 3.2.2 Chemical Stability

Ba_0.5_Sr_0.5_Co_0.8_Fe_0.2_O_3−δ_ (BSCF) is a widely investigated cathode material for intermediate temperature SOFCs (IT‐SOFCs), but its chemical stability is compromised by the acidic oxide CO_2_ in the air on account of its partial composition of alkaline earth metal elements with strong basicity.^[^ ^28^ ^]^ The XRD patterns and long‐term test depicted in Figure 7d,e indicated that BSCF would react with CO_2_ after being exposed to dry air for 50 h at 700 °C, whereas F‐doped BSCF exhibited remarkable stability.

Furthermore, the BaCeO_3_‐based perovskite oxides, which have excellent proton conductivity and are commonly used in protonic ceramic fuel cells (PCFCs) and protonic ceramic electrolysis cells (PCECs), similarly suffer from their high basicity and thus show poor resistance in CO_2_, easily generating BaCO_3_ and CeO_2_.^[^ ^87^ ^−^ ^90^ ^]^ According to the acid‐base conjugation theory, the acidity from HF to HBr increases gradually, while the basicity of F^−^, Cl^−^, and Br^−^ weakens gradually. Furthermore, the basicity of halide ions is weaker than that of oxygen ions. For the purpose of reducing the basicity of BaCeO_3_‐based materials, several materials doped with halogen ions such as BaCe_0.8_Sm_0.2_O_2.9−δ_Cl_0.1_,^[^ ^13^ ^]^ BaCe_0.8_Sm_0.2_O_2.9−δ_F_0.1_ (BCSF),^[^ ^25^ ^]^ BaCe_0.9_Gd_0.1_O_2.9−δ_X_0.1_ (X = F, Cl, Br),^[^ ^46^ ^]^ have been successfully developed. Since the basicity of Ba−X is much weaker than that of Ba−O, the stability of halogen‐doped BaCeO_3_‐based materials in CO_2_ atmosphere could be greatly improved. For example, as demonstrated in Figure 7f,g, the chemical stability of F‐doped BaCe_0.8_Sm_0.2_O_3.0−δ_ (BCS) electrolyte was well optimized. Additionally, based on the investigation results of BaCe_0.9_Gd_0.1_O_2.9−δ_X_0.1_ (X = F, Cl, Br),^[^ ^46^ ^]^ Luo et al. further confirmed that Br‐doping significantly enhanced the stability of BCG in CO_2_ and H_2_O atmosphere without compromising the ionic conductivity.^[^ ^44^ ^]^

#### 3.2.3 Metal‐Oxygen Bonding Strength

One of the most important effects of anion doping on perovskite oxides is regulating the strength of metal−oxygen bond. It is well known that the strength of metal−oxygen bond has immense significance for perovskite oxides, particularly in relation to oxygen vacancies,^[^ ^15^, ^18^, ^30^, ^33^ ^]^ oxygen diffusion properties (oxygen mobility),^[^ ^81^, ^91^ ^]^ alkalinity and carbon deposition resistance,^[^ ^92^ ^]^ etc. Specifically, as for F‐doping, as the electronegativity of F (4.00) is higher than that of O (3.44), F^−^ has a strong electron withdrawing effect, which can reduce the valence electron density of oxygen when it occupies the lattice O‐site, resulting in the weakening of the Coulombic force between B‐site ions and oxygen ion. For example, the projected density of states (PDOS) on metal 3*d* and oxygen 2*p* states of BSCF model and F‐doped BSCF model presented in Figure 7h indicated that F‐doping results in the disappearance or weakening of resonance peaks between O 2*p* and M (Fe and Co) 3*d*, which suggested a weakened M−O hybridization and therefore, weaker bonding interactions between O and M in BSCF.^[^ ^40^ ^]^ Moreover, the activation energy required for oxygen ion dissociation would be lowered; oxygen vacancies were more easily formed; and simultaneously oxygen ion mobility was improved.

Doping with anions of lower electronegativity than O will increase the valence electron density of oxygen, theoretically resulting in an increase in covalency between metal and oxygen.^[^ ^34^, ^93^, ^94^ ^]^ O K‐edge XAS spectra can provide direct insight into the covalent interactions between metal 3*d* and oxygen 2*p* orbitals, as the pre‐edge peak below ≈532 eV corresponds to the unoccupied O 2*p* orbitals that are hybridized with transition metal 3d orbitals. The O K‐edge XAS spectra presented in Figure 7i indicated that the pre‐edge peak in LFOCl exhibited a lower energy position and a higher intensity compared to that of the parent LFO, demonstrating the enhanced Fe−O covalency.^[^ ^34^ ^]^ The case of introducing F into the interlayer gap is consistent with this. According to the theory in the research of Goodenough et al.,^[^ ^85^ ^]^ the active redox pairs located in the highest energy level of O 2*p* band of the B−O molecular orbital model depend on the covalency between B‐site and oxygen. Moreover, the active redox pair of B‐site ions has a larger O 2*p* character, which exerts a more dominant influence on the O 2*p* orbital energy level than A‐site ions. Thus, charge transfer between surface cations and adsorbates (such as O_2_^2−^ and O^2−^) will be promoted in the rate determining steps (RDSs) of OER. As discussed above, the stronger covalency between the B‐site element and oxygen will result in higher OER performance.

## 4 Applications of Anion Doping Perovskite Oxides

Nowadays, researches on anion doping in perovskite oxides are increasing rapidly, making it a novel and potentially effective modification approach. Due to its significant impact on the physical and chemical properties of perovskite oxides, anion doping has become a widely used modification strategy in the field of electrocatalysis. This part will provide a comprehensive overview of its specific applications in this field, focusing on both low‐temperature and high‐temperature perspectives.

### 4.1 Low Temperature Electrocatalysis

At present, the application of anion‐doped perovskite oxides in the field of low temperature mainly focuses on alkaline water electrolysis, solid lithium batteries, zinc‐air batteries and so on.

#### 4.1.1 Alkaline Water Electrolysis

**Figure 8:**
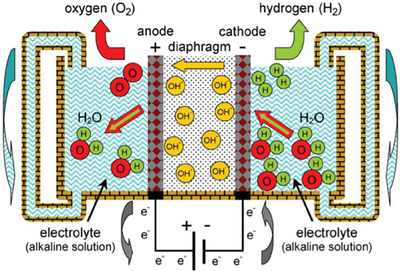
A schematic illustration of a basic alkaline water electrolysis system. Reproduced with permission.^[^ ^21^ ^]^ Copyright 2018, American Institute of Science.

Alkaline water electrolysis (AWE) currently stands as the most well‐established and extensively utilized technology for producing hydrogen on a large scale. Nonetheless, the most significant challenge facing the technology is the cost of the materials used as the electrodes, which are typically made from expensive metals or metal oxides such as IrO_2_ and RuO_2_, due to their excellent catalytic activity for the oxygen evolution reaction (OER), 4OH^−^ = O_2_ + 2H_2_O + 4e^−^, the key rate‐limiting step in the electrochemical process of water splitting.^[^ ^95^ ^]^ The schematic illustration of AWE is shown in **Figure** **8**. Current research efforts are focused on developing alternative electrode materials that are low‐cost and offer similar performance, while perovskite oxides have been developed as candidates for electrode materials, and anion doping has emerged as a novel modification strategy that has gained the attention of many researchers.

**Figure 9:**
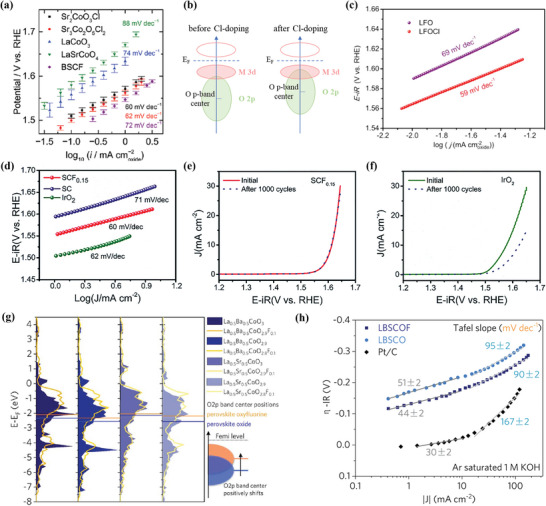
a) OER Tafel plots and Tafel slopes of the catalysts calculated from average currents of positive and negative scans. Reproduced with permission.^[^ ^93^ ^]^ Copyright 2017, Royal Society of Chemistry. b) Schematic diagram of the influence of Cl‐doping on the O 2p‐band center. c) Tafel plots of LFO and LFOCl. Reproduced with permission.^[^ ^34^ ^]^ Copyright 2021, Elsevier. d) Tafel plots of SrCoO_2.85−δ_F_0.15_ (SCF_0.15_), Sr_2_Co_2_O_5_ (SC) and IrO_2_. e) LSV curves of SCF_0.15_ and f) IrO_2_ catalysts initially and after 1000 cycles in 1 m KOH. Reproduced with permission.^[^ ^65^ ^]^ Copyright 2019, Royal Society of Chemistry. g) Projected density of states of O 2p‐bands (sum of the up‐spin and down‐spin states) for La_0.5_Ba_0.5_CoO_2.9−δ_F_0.1_, La_0.5_Ba_0.5_CoO_3−δ_, La_0.5_Sr_0.5_CoO_2.9−δ_F_0.1_, and La_0.5_Sr_0.5_CoO_3−δ_ (δ = 0 or 0.1). h) Tafel plots and slopes for OER in O_2_‐saturated 1 m KOH. Reproduced with permission.^[^ ^86^ ^]^ Copyright 2018, Elsevier.

The R‐P structure Sr−Fe‐based halide perovskite Sr_2_FeO_2_Cl_2_ was investigated for its OER performance in alkaline media.^[^ ^47^ ^]^ To explore the potential for improved OER activity, Miyahara et al. studied the electrocatalytic activity of Sr_2_CoO_3_Cl and Sr_3_Co_2_O_5_Cl_2_ and the effect of Cl‐doping on the activity, as Co ions were generally more active in OER than Fe ions.^[^ ^93^ ^]^ The results showed that the OER Tafel slopes of Sr_2_CoO_3_Cl and Sr_3_Co_2_O_5_Cl_2_ were 60 and 62 mV dec^−1^ respectively, which was equivalent to IrO_2_ (**Figure** **9**a). At the same time, the density functional theory (DFT) calculations also implied that the 2*p*‐band center of O shifted upward closer to the Fermi level due to the introduction of Cl, as shown in Figure 9b.

According to the researches, the OER activity of perovskite oxides could be explained by the difference between the center of the O 2*p*‐band and the Fermi level.^[^ ^96^, ^97^ ^]^ Liu et al. proposed that this difference was associated with the hybridization of metal elements between 3*d*‐orbitals and O 2*p*‐orbitals.^[^ ^98^ ^]^ As the O 2*p*‐band center increased, indicating a higher degree of O 2*p*‐band delocalization, the band gap between O 2*p* and M 3*d* shortened, thereby enhancing OER performance. It was found that the appropriate Cl‐doping in LaFeO_3−δ_ could also improve its OER activity in several aspects such as oxygen vacancy concentration and Fe−O covalency.^[^ ^34^ ^]^ After Cl‐doping, LaFeO_2.9−δ_Cl_0.1_ (LFOCl) obtained 3 times higher intrinsic activity than LaFeO_3−δ_ (LFO), along with a small Tafel slope of 59 mV dec^−1^ (*vs*. LaFeO_3−δ_ 69 mV dec^−1^), as depicted in Figure 9c. IrO_2_ possesses excellent OER activity with a Tafel slope of about 60 mV dec^−1^ (the specific value is subject to the experimental data), which is usually just used as a reference for OER tests because of its high cost. Therefore, it is generally believed that catalysts with OER activity reaching the level of IrO_2_ are full of potential and promising. Similar results could be found on the F‐doped perovskites. For example, Ba_0.5_Sr_0.5_Co_0.8_Fe_0.2_O_3−δ_F_x_ (x = 0.1–0.3)^[^ ^40^ ^]^ and F‐doped SrCoO_2.85−δ_F_0.15_ (SCF_0.15_)^[^ ^65^ ^]^ proved that F doping could also significantly improve the OER activity of perovskites. The cubic phase SrCoO_2.85−δ_F_0.15_ exhibited much higher OER activity than the hexagonal phase Sr_2_Co_2_O_5_, achieving catalytic mass activity 92.72 mA mg^−1^ with an over potential of 420 mV, which was 6 times higher than that of Sr_2_Co_2_O_5_ (15.82 mA mg^−1^) and 26% higher than IrO_2_, along with a Tafel slope of 60 mV dec^−1^ (*vs*. Sr_2_Co_2_O_5_ 71 mV dec^−1^), as shown in Figure 9d.^[^ ^65^ ^]^ More importantly, as demonstrated in Figure 9e,f, SCF_0.15_ still maintained good electrocatalytic performance after 1000 cycles, while a rapid degradation of OER activity occurred on IrO_2_.^[^ ^65^ ^]^ Luo et al. used SSR method to synthesize La_0.5_Ba_0.25_Sr_0.25_CoO_2.9−δ_F_0.1_ (LBSCOF) and found out that F‐doping could promote the proton and electron transfer process by replacing part of O with F and reduce the desorption energy of Co−OO*.^[^ ^86^ ^]^ Meanwhile, this research, as illustrated in Figure 9g, also verified the theory of Yang et al.^[^ ^96^ ^]^ that the introduction of F could shorten the difference between the O 2p‐band center and the Fermi level and promote the formation of mobile oxygen species. Regarding electrocatalysis, the F‐doped LBSCOF exhibited a lower Tafel slope than the substrate, indicating that F‐doping can enhance the OER activity of LBSCO. As shown in Figure 9h while the performance of LBSCO(F) fell short of that of Pt/C at low current density, its catalytic activity overtook above a current density of 20 mA cm^−2^.

#### 4.1.2 Energy Storage Devices

##### Lithium Batteries

Currently, in the field of lithium battery applications, perovskite oxides are commonly employed as ceramic solid electrolytes or lithium‐rich cathode materials. Ceramic electrolytes exhibit superior ionic conductivity compared to polymers, with a potential to reach 10^−4^ S cm^−1^ at room temperature. And lithium‐rich materials make the unit cell shrink, lower the volume change during charging and discharging, and thus improve the structural stability and cycle performance. Anion doping strategies are currently less used in this area and required to be further developed.

**Figure 10:**
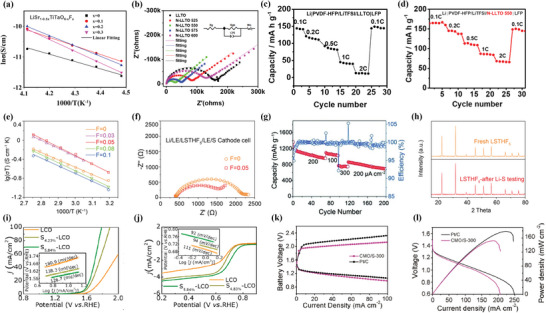
a) Temperature dependencies of total conductivity of LiSr_1−0.5x_TiTaO_6−x_F_x_ samples. Reproduced with permission.^[^ ^43^ ^]^ Copyright 2019, Elsevier. b) Nyquist plots of nitrogen doped LLTO. The inset is the equivalent circuit for EIS. Reproduced with permission.^[^ ^19^ ^]^ Copyright 2021, IOPscience. Corresponding charge‐discharge profiles of c) LLTO and d) N‐LLTO 550. Reproduced with permission.^[^ ^19^ ^]^ Copyright 2021, IOPScience. e) Temperature dependence of Li‐ion conductivities of Li_0.38_Sr_0.44_Ta_0.75−x_Hf_0.25+x_O_3−x_F_x_ with 0 ≤ x ≤ 0.1. f) The EIS of Li‐S battery at 25 °C with Li_0.38_Sr_0.44_Ta_0.75−x_Hf_0.25+x_O_3−x_F_x_ (x = 0 and x = 0.05). g) The cycling performance at different current densities of a Li‐S battery operating at 25 °C. h) XRD results of LSTHF_0.05_ before and after cycling in the Li‐S battery. Reproduced with permission.^[^ ^16^ ^]^ Copyright 2018, John Wiley and Sons. i) The polarization curves and Tafel plots of all catalysts for the OER. j) ORR polarization curves of all samples. Reproduced with permission.^[^ ^61^ ^]^ Copyright 2020, American Chemical Society. k) Voltage‐current polarization data based on CMO/S‐300 and Pt/C catalyst. l) Polarization curves and power densities of primary batteries. Reproduced with permission.^[^ ^17^ ^]^ Copyright 2018, John Wiley and Sons.

Ag_3_MoO_3_F_3_ was the earliest perovskite oxyfluoride as a cathode material used in lithium batteries. This cryolite perovskite could achieve a theoretical specific capacity of 153 mAh g^−1^ (with 3 electron transfers) at a voltage of 1.5 V.^[^ ^11^ ^]^ However, this field has remained largely being neglected for decade, as the timeline showed in Figure 1. In 2019, Sun et al. applied F‐doped perovskite oxide as electrolyte in all‐solid‐state lithium battery.^[^ ^43^ ^]^ Based on previous researches on Li_3x_La_2/3−x_TiO_3_ (LLTO) perovskites and garnet‐type Li_7−σ_La_3_Zr_2−x_Ta_x_O_12_ (LLZTO),^[^ ^99^ ^−^ ^102^ ^]^ Sun et al. prepared F‐doped LiSr_1−0.5x_TiTaO_6−x_F_x_ (LSTTF_x_, x = 0–0.4) by conventional SSR method.^[^ ^43^ ^]^ Electrochemical test in **Figure** **10**a confirmed that when x = 0.1, the sample could reach a higher ionic conductivity of 3.67 × 10^−4^ S cm^−1^ than the parent oxide as 2.90 × 10^−4^ S cm^−1^ at room temperature with migration activation energy of 23.2 kJ mol^−1^, which was comparable to the garnet‐type materials. However, with the increase of F‐doping, the length matching relationship between the A−O and B−O bonds could cause the distortion of the octahedron and block the Li^+^ transport path. Only the conductivity test of the electrolyte was conducted, while the specific battery test was not mentioned. Furthermore, Wu et al. found that N‐doping weakened the bonding of Li ions on the A‐sites in perovskite LLTO structure and allowed for larger lattice distortion, which decreased the activation energy required for Li‐ion hopping.^[^ ^19^ ^]^ As the EIS demonstrated in Figure 10b, the depressed semicircle at high‐to‐intermediate frequencies was attributed to Li^+^ migration in the domain interior and across the domain boundaries and the straight line at low frequencies was ascribed to blocking of Li^+^ at the block electrodes. Furthermore, N‐doped LLTO nanofibers incorporated with a PVDF‐HFP polymer, forming a solid‐state composite electrolyte, demonstrated superior rate capability and cycling stability at room temperature compared to the counterparts with pristine LLTO nanofibers, as presented in Figure 10c,d.^[^ ^19^ ^]^

Moreover, Goodenough et al. developed a new perovskite oxyfluoride Li_0.38_Sr_0.44_Ta_0.7_Hf_0.3_O_2.95_F_0.05_ (LSTHF_0.05_) as Li^+^ solid electrolyte, with a maximum Li‐ion conductivity of 4.8 × 10^−4^ S cm^−1^.^[^ ^16^ ^]^ The ionic conductivity of the samples with different fluorine contents illustrated in Figure 10e confirmed that proper F‐doping is favorable. They further demonstrated excellent cycling performance in not only an all‐solid‐state Li/LiFePO_4_ battery, but also a Li‐S battery with a polymer‐gel cathode, and a supercapacitor. As far as the Li‐S battery was concerned, the total resistance of the Li‐S battery using LSTHF_0.05_ in Figure 10f was 800 Ω cm^2^, which was one‐third of that of the Li‐S battery with the garnet electrolyte LLZTO.^[^ ^103^ ^]^ According to Figure 10g, during the following 200 cycles, the battery maintained a high Coulombic efficiency close to 100%, indicating that LSTHF_0.05_ successfully blocked the polysulfide shuttle, a prevalent issue in current battery technology. Moreover, the reversible cycle capacity remained stable at ≈975 mAh g^−1^ after 100 cycles, with 90.7% of the stable capacity retained in the second cycle. XRD profiles depicted in Figure 10h indicated that LSTHF_0.05_ had excellent stability in Li‐S batteries.

Currently, the utilization of perovskite oxides, modified through anion doping strategies, is primarily focused on solid‐state electrolytes for lithium batteries. In fact, perovskite oxides have been widely used as cathode materials in Li‐ion batteries, Li‐S batteries, and Li‐air batteries.^[^ ^104^ ^−^ ^107^ ^]^ Therefore, this modification strategy holds tremendous potential in the field of lithium batteries. Furthermore, delving into the corresponding mechanism behind this modification can significantly advance the development of structure‐property relationships.

##### Zinc‐Air Batteries

Zinc‐air batteries have an ideal energy density and power density, and are anticipated to be widely used in the field of energy conversion and storage.^[^ ^108^ ^−^ ^110^ ^]^ Thereinto, the air electrode is the core area of the oxygen catalytic reaction and even the focal point in the entire zinc‐air battery research. For perovskite oxides used in zinc‐air batteries, F^−^ and S^2−^ are mainly used to replace part of O^2−^ to regulate the electronic structure.

Air electrode with excellent ORR/OER bifunctional catalytic activity is very critical for zinc‐air batteries.^[^ ^111^ ^]^ Based on the advantage of anion doping on ORR/OER performance, it is a promising way to modify the air electrode using anion doping. S‐doping has been utilized to improve ORR/OER performance of LaCoO_3_ (LCO).^[^ ^61^ ^]^ The results in Figure 10i demonstrated that S_5.84%_‐LCO exhibited superior OER electrocatalytic performance compared to the undoped, with a lower overpotential of 364 mV (10 mA cm^−2^) and a lower Tafel slope of 126.7 mV dec^−1^, showing the best OER electrocatalytic performance among them. As for ORR activity, in Figure 10j, S_5.84%_‐LCO possessed a higher limiting current density of 4.8 mA cm^−2^ at 0.2 V than S_4.23%_‐LCO (4.5 mA cm^−2^ at 0.2 V) and LCO (4 mA cm^−2^ at 0.2 V). The same lower Tafel slope indicated that S_5.84%_‐LCO has better ORR electrocatalytic performance.^[^ ^61^ ^]^ Furthermore, the total activity ΔE (= E_OER_−E_ORR_) calculated from the potential of OER (10 mA cm^−2^) and the half‐wave potential of ORR confirmed that S_5.84%_‐LCO showed the smallest ΔE value (0.89 V), indicating S_5.84%_‐LCO had the best ORR/OER catalytic performance. After S‐doping optimization, S_5.84%_‐LCO obtained a higher power density of 92 mW cm^−2^ at 144 mA cm^−2^ than that for LCO (39 mW cm^−2^ at 63 mA cm^−2^).

Similarly, Peng et al. utilized the electrospinning method to synthesize S‐doped CaMnO_3_ nanotubes (CMO/S) followed by thermal calcination and vulcanization treatment, and achieving precise control over the sulfur content and oxygen vacancies in CMO through varying calcination temperatures.^[^ ^17^ ^]^ DFT was used to calculate the state density pre‐ and post‐vulcanization, with a negative shift in both the valence and conduction bands. This phenomenon could be regarded as the lower electronegativity of S compared to O, which could enhance the M−O bond covalent and ultimately shorten the band gap. Moreover, the Tafel slope of the CMO/S electrode sintered at 300 °C was only 52 mV dec^−1^, smaller than that of the commercial Pt/C electrode as 62 mV dec^−1^, which meant that it had a larger current density under low overpotential (as illustrated in Figure 10k) and exhibited exceptional OER performance. The peak power density, as shown in Figure 10l, reached 152 mW cm^−2^ at 0.82 V, which was comparable to that of Pt/C. S‐doping, as an anion‐doping strategy, is relatively less utilized but has a dual function of enhancing conductivity and surface vacancy defects. However, its practical implementation is confined to low temperature due to the volatility of the S element at high temperatures.

##### Other Fields and Perspective

In addition to the energy storage devices mentioned above, there is a number of literatures on perovskite oxides as electrode materials for Na‐ion batteries^[^ ^112^ ^−^ ^114^ ^]^ and super capacitors.^[^ ^115^ ^−^ ^117^ ^]^ The modification of these oxides using anions such as F, Cl, S, and N shows great promising. Moreover, for Li‐S/Na‐S batteries, the S‐doping strategy may have a more pronounced impact. For instance, Zhang et al. reported the application of Ba_0.5_Sr_0.5_Co_0.8_Fe_0.2_O_3−δ_ in Li‐S batteries and demonstrated that BSCF favored the dual‐bonding (Li−O and Sr−S bonds) to anchor lithium polysulfides,^[^ ^118^ ^]^ which enhanced their interfacial affinity on the perovskite host and induces Li_2_S deposition. Based on this research, if S^2−^ could be doped into the perovskite oxides, it may further enhance the deposition of Li_2_S and the immobilization of lithium polysulfide, while regulating the electronic structure of high‐valent Co and Fe ions.

### 4.2 High Temperature Electrocatalysis

Regarding high temperature circumstance, N‐doping and S‐doping strategies are not suitable due to their volatileness. Currently, at high temperatures, perovskite oxides are mainly applied for the electrode materials of solid oxide cells.

#### 4.2.1 Oxygen‐Ion Conducting Solid Oxide Cells

**Figure 11:**
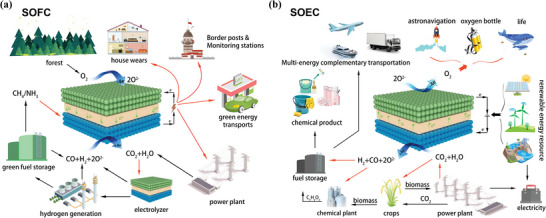
Applicational schematic diagram of oxygen‐ion conducting solid oxide cells. a) SOFC, b) SOEC. Reproduced with permission.^[^ ^23^ ^]^ Copyright 2022, Elsevier.

SOFC is a type of environmentally friendly energy conversion device that can directly convert the chemical energy stored in fuel and oxidizers into electrical energy. Conversely, the SOEC is the reverse process of the SOFC and can directly convert electrical energy generated by clean sources such as solar and wind power into chemical energy for transportation and storage.^[^ ^119^ ^]^ The applicational schematic diagrams of SOFC and SOEC are shown in **Figure** **11**a,b, respectively.

##### Solid Oxide Fuel Cells

**Figure 12:**
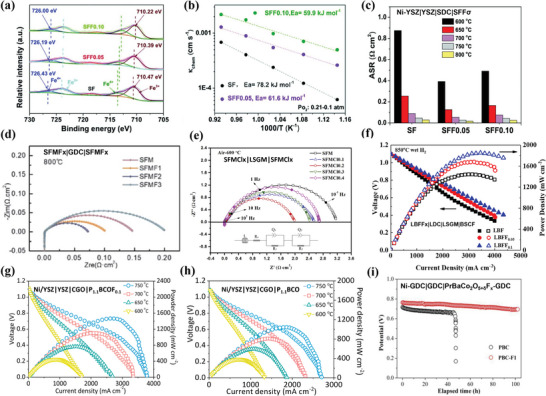
a) XPS of Fe 2p peaks for the SFF_σ_ samples. b) Oxygen surface reaction rate constant *k*_chem_ of the SFF_σ_ versus 1000/T. c) ASR values of the SFF_σ_ samples. Reproduced with permission.^[^ ^15^ ^]^ Copyright 2017, John Wiley and Sons. d) the EIS spectra of SFMFx air electrodes under open‐circuit conditions. Reproduced with permission.^[^ ^33^ ^]^ Copyright 2020, Royal Society of Chemistry. e) the EIS of the SFMClx air electrodes. Reproduced with permission.^[^ ^32^ ^]^ Copyright 2020, Elsevier. f) *I−V−P* curves of single cells with La_0.5_Ba_0.5_FeO_3−x−δ_F_x_ (x = 0, 0.05, 0.1) fuel electrodes at 850 °C. Reproduced with permission.^[^ ^39^ ^]^ Copyright 2022, Elsevier. *I−V−P* curves of g) P_1.1_BCOF_0.1_ air electrode and h) P_1.1_BCO air electrode. Reproduced with permission.^[^ ^66^ ^]^ Copyright 2023, Elsevier. i) Cell potential versus elapsed time for PBC and PBC‐F1 single cells. Reproduced with permission.^[^ ^26^ ^]^ Copyright 2018, Elsevier.

F‐doped SrFeO_3−δ_ (SFF_σ_, σ = 0, 0.05, 0.1) was studied as SOFC air electrode. Compared with the conventional SOFC operating temperature (800–850°C), the use of SFF_σ_ air electrodes could greatly reduce the operating temperature.^[^ ^15^ ^]^ As presented in **Figure** **12**a, through XPS analysis of the Fe‐2*p* peak, it was found that the binding energy of Fe^4+^ and Fe^3+^ gradually decreased with F‐doping, and the binding energy of Sr‐3*d* was also observed a similar phenomenon. This implied that the introduction of F^−^ could reduce the binding energy of metal and oxygen, thereby increasing the chemical diffusion coefficient and surface oxygen exchange coefficient (Figure 12b). SrFeO_2.95−δ_F_0.05_ showed the best area specific resistance (ASR) of 0.393 Ω cm^2^ at 600 °C, which was less than half of that for SrFeO_3−δ_ as 0.875 Ω cm^2^ and comparable to many A‐site or B‐site cation‐doped SrFeO_3−δ_ electrodes, as shown in Figure 12c.^[^ ^15^ ^]^

Double perovskite Sr_2_Fe_1.5_Mo_0.5_O_6−x−δ_F_x_ (SFMF_x_, x = 0, 0.1, 0.2 and 0.3) was also investigated as SOFC air electrode.^[^ ^33^ ^]^ It was found that SFMF_0.2_ possessed a minimum ASR of 0.072 Ω cm^2^ at 800 °C, which was half of the undoped SFM and exhibited the lowest polarization resistance at different temperature, as presented in Figure 12d. The peak power density of SFM and SFMF_0.2_ achieved at 800°C was found to be 0.418 and 0.534 W cm^−2^. In addition, Cl‐doping was also adopted to SFM. Sr_2_Fe_1.5_Mo_0.5_O_6−x−δ_Cl_x_ (SFMCl_x_, x = 0, 0.1, 0.2, 0.3, 0.4) had been applied to SOFC air electrode materials,^[^ ^32^ ^]^ which also showed lower polarization impedance than SFM (Figure 12e). It is worth noticing that these anion‐doping materials all reflect lower catalytic performance (higher polarization resistance) when the amount of anion‐doping is higher. The reason is that the oxygen vacancy concentration will decrease with the increase of anion‐doping,^[^ ^15^ ^]^ which will affect the process of oxygen reduction and migration.

Apart from Sr−Fe‐based perovskites oxides, anion doping is also applied in other system. For instance, La_0.6_Sr_0.4_Co_0.2_Fe_0.8_O_3−δ−x_F_x_ (LSCFF_x_, x = 0, 0.05, 0.1) was utilized as an air electrode for SOFC in a fuel electrode‐supported Ni−YSZ system.^[^ ^27^ ^]^ The best ASR 0.017 Ω cm^2^ was achieved at x = 0.05, and the peak power density (PPD) of the single cell reached 1.00 W cm^−2^ at 850 °C, which was 33% higher than that of LSCF. In another study, a single cell supported by a 300 µm‐thick La_0.8_Sr_0.2_Ga_0.8_Mg_0.2_O_3−δ_ electrolyte layer with La_0.5_Ba_0.5_FeO_3−δ_ fuel electrode showed a PPD of 1.45 W cm^−2^ at 850 °C, which increased to 1.86 W cm^−2^ when La_0.5_Ba_0.5_FeO_2.9−δ_F_0.1_ was substituted, as shown in Figure 12f.^[^ ^39^ ^]^

Moreover, F‐doping can not only enhance the electrocatalytic performance but also improve the stability of cobalt‐based materials system. Zhao et al. found that the F‐doped Pr_1.1_Ba_0.9_Co_2_O_5+δ_F_0.1_ (P_1.1_BCOF_0.1_) air electrode delivered a PPD of 1.10 W cm^−2^ at 700 °C in the fuel electrode‐supported Ni−YSZ system, which was higher than that of Pr_1.1_Ba_0.9_Co_2_O_5+δ_ (P_1.1_BCO) as 0.812 W cm^−2^, as demonstrated in Figure 12g,h.^[^ ^66^ ^]^ As known, cobalt‐based materials have excellent electrocatalytic activity but are limited by their higher CTE compared to the buffer layer or the electrolyte, which seriously impedes its long‐term stability. Wan et al. demonstrated that F‐doped PrBaCo_2_O_5+δ_ showed better durability in 100 h test than that of the undoped one, as shown in Figure 12i.^[^ ^26^ ^]^

##### Solid Oxide Electrolysis Cells

**Figure 13:**
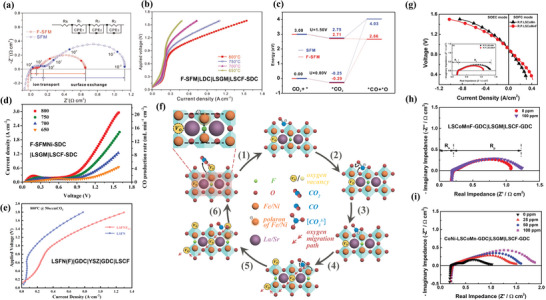
a) Impedance spectrum of F‐SFM and SFM at 800 °C. b) *I−V* curves of single‐cell performance for pure CO_2_ electrolysis using F‐SFM fuel electrode at 650–800 °C. c) The relative energy profile for CO_2_ adsorption and disassociation reaction. Reproduced with permission.^[^ ^18^ ^]^ Copyright 2018, John Wiley and Sons. d) *I−V* curves of a single cell with the F‐SFMNi−SDC fuel electrode for pure CO_2_ electrolysis at 650–800 °C. Reproduced with permission.^[^ ^82^ ^]^ Copyright 2022, American Chemical Society. e) *I−V* polarization curve of LSFNF_0.1_ from OCV to 1.8 V. f) CO_2_ electrolysis reaction mechanism for the F‐doped LSFNF_0.1_. Reproduced with permission.^[^ ^37^ ^]^ Copyright 2022, American Chemical Society.(g) *I−V* curves of the single cell with LSCoMn and LSCoMnF for the CO_2_ electrolysis and the corresponding EIS curves. h) EIS curves of the single cell with LSCoMnF under the reaction gas (30% CO/CO_2_) streams that containing 100 ppm H_2_S. Reproduced with permission.^[^ ^30^ ^]^ Copyright 2020, American Chemical Society. i) EIS curves of the single cell with CoNi−LSCoMn using the reaction gas streams that contain different concentrations of H_2_S. Reproduced with permission.^[^ ^120^ ^]^ Copyright 2020, Royal Society of Chemistry.

As regard to SOEC, Sr_2_Fe_1.5_Mo_0.5_O_6−δ_F_0.1_ (F‐SFM) was also used as SOEC fuel electrode material for CO_2_ electrolysis. In the symmetrical cell test, the polarization impedance of 0.656 Ω cm^2^ for F‐SFM was much lower than 1.130 Ω cm^2^ for SFM in 1:1 CO‐CO_2_ at 800 °C, as exhibited in **Figure** **13**a. In full cell test, the cell could obtain pure CO_2_ electrolysis current density of 1.36 A cm^−2^ at 1.5 V and 800 °C (Figure 13b), almost double of 0.71 A cm^−2^ for SFM, reflecting better CO_2_‐RR performance.^[^ ^18^ ^]^ Theoretical computation of the relative energy profiles is depicted in Figure 13c, where “*CO_2_” and “*CO+*O” are represented to CO_2_ adsorption and dissociation reactions, respectively.^[^ ^18^ ^]^ The results indicated that when 1.5 V voltage was applied, CO_2_ dissociation via F‐SFM was significantly more energetically favorable than SFM, with exothermic (−0.05 eV) F‐SFM but endothermic (+1.28 eV) SFM, which demonstrated essential performance improvement by F‐doping. F‐doping strategy could also be combined with other modification methods. Xia et al. reported Sr_1.9_Fe_1.5_Mo_0.4_Ni_0.1_O_6−δ_F_0.1_ (SFMNi‐F) with A‐site deficiency as fuel electrode for CO_2_ electrolysis.^[^ ^82^ ^]^ The SFMNi‐F fuel electrode was first treated in 30%H_2_–70%Ar at 700 °C, and then applied for pure CO_2_ electrolysis. The F‐doped SFMNi with in situ exsolved Ni−Fe nanoparticles demonstrated superior performance compared to SFM, achieving a current density of 2.66 A cm^−2^ at 800 °C, 1.5 V for pure CO_2_, as exhibited in Figure 13d.

Besides, single perovskite La_0.6_Sr_0.4_Fe_0.8_Ni_0.2_O_2.9−δ_F_0.1_ (LSFNF_0.1_) was also found more impressive than the undoped for 1.8 times current density as 0.9 A cm^−2^ for CO_2_ electrolysis at 1.5 V and 800 °C, as presented in Figure 13e.^[^ ^37^ ^]^ The effect of F‐doping on the CO_2_ electrolysis mechanism is illustrated in Figure 13f. The electronegativity of F promoted the formation of the polarons, making CO_2_ molecules easy to be activated. Additionally, F‐doping weakened the M−O bond, which mainly accelerated step 2 and 3. Thus, the ambient lattice oxygen ions combined with the adsorbed activated CO_2_ more easily, which consequently increased the concentration of the bidentate carbonate and ultimately promote the electrolysis process. Xia et al. also demonstrated the enhancement of F‐doping on the similar perovskite oxide La_0.5_Sr_0.5_FeO_2.9−δ_.^[^ ^38^ ^]^

F‐doping is also conducive to resist sulfur poison in CO_2_ electrolysis, which is able to be applied in the electrolysis of sulfur containing CO_2_ gas stream. Park et al. adopted F‐doping R‐P perovskite La_0.9_Sr_0.8_Co_0.4_Mn_0.6_O_3.9−δ_F_0.1_ (LSCoMnF) in CO_2_ electrolysis as a SOEC fuel electrode,^[^ ^30^ ^]^ which presented higher performance of 0.499 A cm^−2^ at 1.3 V, 850 °C and lower polarization resistance of 0.853 Ω cm^2^ than the undoped with 0.401 A cm^−2^ and 1.106 Ω cm^2^ (Figure 13g), and little performance degradation was further observed in the long‐term test under H_2_S containing CO_2_ atmosphere.^[^ ^30^ ^]^ Moreover, in their another work, La_1.2_Sr_0.8_Co_0.4_Mn_0.6_O_4_ with in‐situ exsolved Co−Ni alloy nanoparticles (CoNi−LSCoMn) was utilized as sulfur‐tolerant fuel electrode for CO_2_ electrolysis.^[^ ^120^ ^]^ As shown in Figure 13h,i, F‐doping exhibited more advantageous in 100 ppm H_2_S than the in situ exsolution strategy, which could be attributed to the lower basicity of F^−^ than O^2−^. After F‐doping, the basicity of the perovskites would be reduced, thereby improving their chemical stability in acidic atmospheres. Although the current density still needs to be improved compared to many other SOEC reports, the actual application of LSCoMnF is promising and significant for its great tolerance to H_2_S and CO_2_.

Overall, F is the only element with higher electronegativity than O, making F‐doping a crucial strategy in regulating the properties of perovskite oxides, which is the reason that most of the aforementioned high‐temperature electrocatalysis studies adopt F‐doping strategy. The above studies have demonstrated that F‐ and Cl‐doping can significantly improve the performance of perovskite family (single, double and R‐P type) in SOFC/SOEC field, including direct catalytic performance, stability and thermal expansion, etc. However, there are few high‐temperature electrocatalytic studies on perovskite oxides using Cl‐ or Br‐doping strategy, perhaps due to the limited stability of the material caused by the volatility of these elements. Nonetheless, given that Cl‐ and Br‐doping could enhance the covalency of M−O, they hold great promise for applications at intermediate or low temperatures below 700 °C, particularly in air electrode materials related to oxygen electrocatalysis.

#### 4.2.2 Proton‐Conducting Solid Oxide Cells

**Figure 14:**
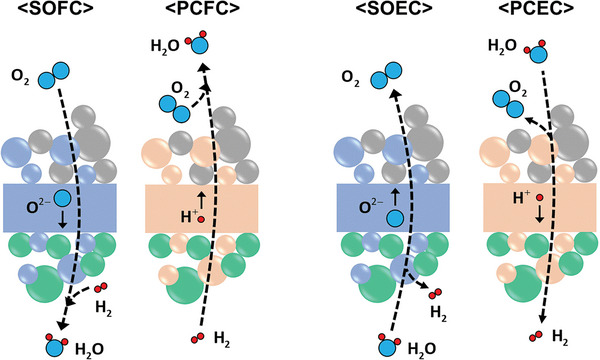
Schematic illustrations of SOFC, PCFC, SOEC, and PCEC. Reproduced with permission.^[^ ^121^ ^]^ Copyright 2020, Springer Nature.

Originating from oxygen ion‐conducting solid oxide cells (O‐SOC), PCFC/PCEC adopts proton conductors as the electrolyte. It has a similar but different electrocatalytic principle compared to SOFC/SOEC, as illustrated in **Figure** **14**. PCFC/PCEC shows great promising in hydrogen related green energy industry (H_2_, NH_3_ and H_2_O) on account of its unique affinity to proton. Moreover, by virtue of high electronegativity of F element, and the presence of H−F hydrogen bonds, F‐doping shows great potential in hydrogen species capture.

As mentioned in Section 3.2.2 above, although Ba‐based cerate has high proton conductivity which is ideal proton conductor electrolyte material. However, its chemical stability is easily affected by the acid gas CO_2_ in the air. Halogen doping can reduce the basicity of perovskite oxides without sacrificing their proton conductivity, thereby effectively inhibiting their carbonation.^[^ ^13^, ^25^, ^44^ ^−^ ^46^, ^122^ ^]^ However, Xie et al. found out that F^−^ in the electrolyte BaCe_0.8_Sm_0.2_F_0.1_O_2.85_ would diffuse to the cathode during operation, which reduced the stability of the electrolytes.^[^ ^28^ ^]^ To solve this problem, F‐doped Ba_0.5_Sr_0.5_Co_0.8_Fe_0.2_O_3−δ_ was developed as a potential cathode for PCFC and efficiently improved the long‐term stability, as evidenced by Figure 7e. Thus, when the halogenated perovskite oxide electrolyte material is used, the diffusion of halogens also needs to be considered.

Since protons are not intrinsic to the lattice of perovskite oxides, their proton conduction is strongly dependent on hydroxide defects formed by the hydration reaction ($V_{O}^{••}$(g) + H_2_O + $O_{O}^{\times }$→2${\mathrm{OH}}_{O}^{•}$) of H_2_O combined at oxygen vacancies.^[^ ^123^, ^124^ ^]^ Therefore, in order to promote the protonic conductivity of perovskite oxides, many studies have been devoted to improving their hydration ability.

**Figure 15:**
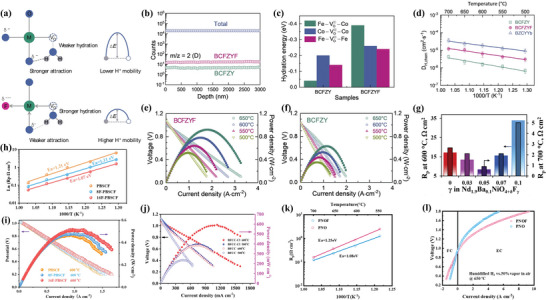
a) Schematic of F‐doping inductive effect to promote the hydration reaction and the proton mobility in perovskite oxides. b) TOF‐SIMS depth profile of m/z = 2 (D) signal in D_2_O treated BCFZY(F) samples. c) Hydration energy for BCFZY(F). d) Arrhenius plots of D_H, chem_ of BCFZY(F) and BZCYYb bar samples. e,f) *I−V−P* curves of PCFC with BCFZY(F) as cathode. Reproduced with permission.^[^ ^125^ ^]^ Copyright 2022, Elsevier. g) Overall polarization resistance versus fluorine content. Reproduced with permission.^[^ ^48^ ^]^ Copyright 2020, Elsevier. h) Arrhenius plots of polarization resistances of F‐doped PBSCF. i) *I−V−P* curves of the single cells using F‐doped PBSCF cathodes. Reproduced with permission.^[^ ^79^ ^]^ Copyright 2022, Elsevier. j) *I−V−P* curves of the BFCC‐based cell, and of the BFCC‐Cl‐based cell measured at 600 and 500 °C, respectively. Reproduced with permission.^[^ ^126^ ^]^ Copyright 2019, Elsevier. k) Arrhenius plots of R_p_ values of PNO and PNOF electrodes. l) *I−V* curves of cathode‐supported single cell at different temperatures. Reproduced with permission.^[^ ^35^ ^]^ Copyright 2021, Elsevier.

Significant role of F‐doping has been proven in promoting the hydration capacity of BaCo_0.4_Fe_0.4_Zr_0.1_Y_0.1_O_2.9−δ_F_0.1_ (BCFZYF).^[^ ^125^ ^]^ Owing to higher electronegativity of F^−^ compared to O^2−^, F‐doping would enhance the polarity of M−$V_{O}^{••}$−M, therefore promoted adsorption of H_2_O on oxygen vacancies to form more protonic defects. Meanwhile, as the schematic illustrated in **Figure** **15**a, F^−^ could reduce the negative charge of lattice oxygen, leading to weaker O**···**H interactions, thereby lowering the diffusion barrier of proton in the perovskite oxides. Time of Flight−Secondary Ion Mass Spectrometer (TOF‐SIMS) was applied to observe the distribution of D element in BCFZY(F) samples which were treated by D_2_O (pD_2_O = 10% atm) at 500 °C for 24 h, as shown in Figure 15b, the D signal in BCFZYF sample was obviously stronger than that of BCFZY.^[^ ^125^ ^]^ Moreover, DFT calculation in Figure 15c demonstrated that the hydration energies for BCFZYF were much lower than that of the BCFZY in three vacancy models, including Fe−$V_{O}^{••}$−Co, Co−$V_{O}^{••}$−Co, and Fe−$V_{O}^{••}$−Fe.^[^ ^125^ ^]^ The proton mobility of BCFZY was also promoted by F‐doping, as indicated in Figure 15d, D_H, chem_ of BCFZYF was 1.21 × 10^−5^ cm^2^ s^−1^ at 700 °C, which was three times that of BCFZY (4.30 × 10^−6^ cm^2^ s^−1^) and close to the electrolyte BaZr_0.1_Ce_0.7_Y_0.1_Yb_0.1_O_3−δ_ (BZCYYb). The *I−V−P* curves of Ni−BZCYYb‐supported PCFC with BCFZY(F) as air electrode were shown in Figure 15e–f, where PPD of 0.921 and 0.631 W cm^−2^ were obtained at 650 °C in BCFZYF‐based cell and BCFZY‐based cell, respectively.^[^ ^125^ ^]^ In another work of PrBa_0.5_Sr_0.5_Co_1.5_Fe_0.5_O_6−δ_ (PBSCF), Xu and coworkers confirmed that F‐doping was conducive to the proton involved oxygen reduction reaction (P−ORR),^[^ ^79^ ^]^ as shown in Figure 15h, the Arrhenius plots of PBSCF, 8F‐PBSCF, and 16F‐PBSCF electrodes indicated that 16F−PBSCF possessed the lowest polarization resistances and activation energy. Figure 15i exhibited the comparison of *I−V−P* curves of Ni−BZCYYb‐supported PCFC with these air electrodes, 16F‐ PBSCF obtained a higher PPD of 0.51 W cm^−2^ than the undoped as 0.46 W cm^−2^ at 600 °C.^[^ ^79^ ^]^ Regrettably, the authors did not specifically analyze the mechanism of F‐doping in the P‐ORR.

Besides, an anion and cation co‐doped strategy was adopted in BaFeO_3−δ_, in which F^−^, Ca^2+^, Sn^4+^ and Y^3+^ were co‐doped as Ba_0.95_Ca_0.05_Fe_0.85_Sn_0.05_Y_0.1_O_2.9−δ_F_0.1_ (BCFSYF_0.1_) for the air electrode of PCFC.^[^ ^29^ ^]^ And it was calculated that F‐doping can reduce the activation energy required for migration of oxygen ions, thus enhanced the electrical conductivity, lowers the polarization resistance and increased the power density of BCFSY from 0.949 W cm^−2^ to 1.05 W cm^−2^ at 700 °C.

Cl‐doping strategy was also favored by researchers. perovskite oxychloride BaFe_0.6_Co_0.3_Ce_0.1_O_2.95−δ_Cl_0.05_ (BFCC‐Cl) was applied for the Ni−BZCYYb‐supported PCFC,^[^ ^126^ ^]^ as presented in Figure 15j, the PPD was improved from 0.386 to 0.593 W cm^−2^ at 600 °C. Due to the opposite difference in electronegativity, the effect of Cl^−^ on the hydration reaction should be quite different from that of F^−^, and the mechanism of Cl‐doping should be further studied.

In PCECs, a Ba‐containing with anion‐doping R‐P‐type material Nd_1.9_Ba_0.1_NiO_4+δ_F_γ_ (γ = 0, 0.03, 0.05, 0.07, 0.1) was proposed as air/steam electrode.^[^ ^48^ ^]^ The highest ionic conductivity and the lowest electrode polarization resistance was found for the composition with γ = 0.05, as shown in Figure 15g, which could be attributed to the mixed anion effect. The current density of the PCEC could achieve 1.37 A cm^−2^ in the thermo‐neutral voltage (U ≈ 1.3 V) at 700 °C, which was comparable with the best results reported for advanced PCECs.

Reversible protonic ceramic cells (RPCCs) show increasing potential for alleviating the global energy crisis and environmental pollution by efficient conversion between hydrogen and electricity. The anion‐doping strategy was also adopted in RPCCs. Li et al. found that F‐doped Pr_2_NiO_4+δ_ (PNO) possessed excellent electrochemical activity and sufficient durability as air electrode.^[^ ^35^ ^]^ In the BCZYYb supported symmetric cell, Pr_2_NiO_3.9+δ_F_0.1_ (PNOF) showed lower polarization resistance and activation energy than the PNO, indicating PNOF possessed better kinetics at lower temperatures (Figure 15k). As presented in Figure 15l, PNOF could achieve a PPD of 0.580 W cm^−2^ in fuel cell mode with humidified H_2_ fuel, while the PNO was 0.320 W cm^−2^, at 650 °C. It was particularly exciting that a current density of 2 A cm^−2^ in the electrolysis cell mode with 50% H_2_O+50% air was obtained at 1.3 V and 650 °C, which is 66.7% higher than that of Pr_2_NiO_4+δ_. Additionally, PNOF also possessed favorable durability in fuel cell mode long‐term test. Under a constant discharge current density of 0.7 A cm^−2^, PNOF obtained about 0.7 V discharge voltage without apparent degradation for 200 h at 650 °C. However, the authors did not show the cycle performance of FC/EC mode for the PNOF electrode, which is also crucial standard to evaluate the P‐ORR and P‐OER activity of the electrode.

## 5 Prospects and Challenges

So far, perovskite oxides modified by anion doping strategy have shown great application prospects in the field of electrocatalysis, in order to further promote this strategy, several prospects for the future research are proposed as follows:

1. It would be promising to apply F, Cl‐doping strategy in electrospinning method, by which the prepared nanofibers show great potential in low and intermediate temperature electrocatalysis, as our previous work proved that La_0.6_Ca_0.4_Fe_0.8_Ni_0.2_O_3−δ_ nanofibers show impressive performance in the med‐temperature SOFC.^[^ ^127^ ^]^ Meanwhile, F‐doped La_0.6_Sr_0.4_Fe_0.8_Ni_0.2_O_2.9−δ_F_0.1_ also shows competitive CO_2_‐RR catalytic activity in SOEC.^[^ ^37^ ^]^ We believe that the anion‐doping strategy will further expand the application of nanofibrous materials in low to medium temperature environments. In addition, the topological chemistry method holds great promising in resolving the doping amount problem in N, S‐doping by quantifying unstable intermediates prior to doping, which is also worth investigating for the synthesis of bromine‐doped perovskite oxides.
2. As anion doping involves the replacement of oxygen ions, it is often more complicated compared to cation doping, particularly in oxygen electrocatalytic reactions. Therefore, it becomes imperative to delve deeper into the mechanisms underlying oxygen ion migration affected by anion doping. To achieve a comprehensive understanding, the integration of DFT calculations with various in situ physical and chemical characterization techniques becomes essential. These techniques encompass in‐situ infrared and Raman spectroscopy, in situ XPS, XRD, XAS, as well as transmission electron microscopy, among others. By employing these methods, it becomes possible to observe the behavior of the doped anions in perovskite oxides, both before and after catalytic testing, which will offer valuable guidance for further exploration of the mechanisms underlying anion doping. Besides, few researches explore whether these doped anions can exist stably in various applications, which also cries out for in situ characterization techniques.
3. By synergistically combining various contemporary modification strategies, such as cation doping, infiltration, and in situ exsolution, it becomes possible to finely tune the structure and non‐stoichiometry of perovskite oxides and other promising candidates. These modifications facilitate the introduction of active interfaces, thereby enhancing the material's functionality and promoting electrocatalytic reactions. Moreover, the scope of perovskite oxides can be extended to all oxides, and big data screening can be used to assist the research of anion doping in different electrocatalytic fields.

## 6 Conclusion

This review presents an overview of the recent advancements in anion oxygen‐site doped perovskite oxides and their applications in electrocatalysis. Anion doping plays a crucial role in fine‐tuning various properties of perovskite oxides, such as lattice structure, oxygen vacancy concentration, electron configuration, basicity, and M─O bond strength. These adjustments have a direct impact on the electrocatalytic activity of the perovskite oxides. It is worth mentioning that F‐doping has been more widely studied than other types of anion doping. The main reason for this is that F is the only element possessing stronger electronegativity than O, coupled with its smaller ionic radius than oxygen ions. Therefore, F‐doping offers distinct advantages over other types of anion doping when it comes to adjusting oxygen vacancies and facilitating oxygen ion migration. However, despite significant progress, the precise mechanisms underlying certain anion doping strategies remain unclear. To address this knowledge gap and facilitate targeted material optimization for specific applications, in‐situ characterization techniques and DFT calculations are urgently required. This comprehensive review aims to provide insights, ideas, and prospects for future anion doping strategies and their subsequent development.

## Conflict of Interest

The authors declare no conflict of interest.
